# Cotton miR393-TIR1 Module Regulates Plant Defense Against *Verticillium dahliae* via Auxin Perception and Signaling

**DOI:** 10.3389/fpls.2022.888703

**Published:** 2022-05-03

**Authors:** Gege Shi, Saisai Wang, Peng Wang, Jingjing Zhan, Ye Tang, Ge Zhao, Fuguang Li, Xiaoyang Ge, Jiahe Wu

**Affiliations:** ^1^Zhengzhou Research Base, State Key Laboratory of Cotton Biology, School of Agricultural Sciences, Zhengzhou University, Zhengzhou, China; ^2^State Key Laboratory of Cotton Biology, Institute of Cotton Research of Chinese Academy of Agricultural Sciences, Anyang, China; ^3^The State Key Laboratory of Plant Genomics, Institute of Microbiology, Chinese Academy of Sciences, Beijing, China

**Keywords:** *Gossypium hirsutum*, *Verticillium dahliae*, GhTIR1, auxin, salicylic acid

## Abstract

Plant auxin is essential in plant growth and development. However, the molecular mechanisms of auxin involvement in plant immunity are unclear. Here, we addressed the function of the cotton (*Gossypium hirsutum*) miR393-TIR1 module in plant defense against *Verticillium dahliae* infection via auxin perception and signaling. *GhTIR1* was directedly cleaved by ghr-miR393 according to mRNA degradome data, 5′-RACE analysis, and a GUS reporter assay. Ghr-miR393 knockdown significantly increased plant susceptibility to *V. dahliae* compared to the control, while ghr-miR393 overexpression and *GhTIR1* knockdown significantly increased plant resistance. External indole-3-acetic acid (IAA) application significantly enhanced susceptibility to *V. dahliae* in ghr-miR393 knockdown and control plants compared to mock treatment, and only slightly increased susceptibility in overexpressing ghr-miR393 and GhTIR1-silenced plants. Application of external PEO-IAA (an auxin antagonist) had a contrary trend with IAA application. Based on yeast two-hybrid and bimolecular fluorescence complementation assays, GhTIR1 interacted with GhIAA14 in the nucleus, and *GhIAA14* knockdown reduced plant resistance to *V. dahliae* infection. The results suggested that the ghr-miR393-GhTIR1 module regulates plant defense via auxin perception and signaling. Additionally, simultaneous knockdown of *GhTIR1* and *GhICS1* significantly increased plant susceptibility to *V. dahliae* compared to the control, indicating that salicylic acid (SA) accumulation is vital for the ghr-miR393-GhTIR1 module to regulates plant resistance. Transcriptome data also demonstrated that *GhTIR1* knockdown significantly downregulated expression of auxin-related genes and upregulated expression of SA-related genes. Overall, the ghr-miR393-GhTIR1 module participates in plant response to *V. dahliae* infection via IAA perception and signaling partially depending on the SA defense pathway.

## Introduction

Auxin has long been reported as an important virulence factor for root gall formation by *Agrobacterium* pathogens (Spaepen and Vanderleyden, 2011). When exogenous auxin was applied at pathogen inoculation, treated plants showed serious disease symptoms due to suppression of salicylic acid (SA)-mediated defenses (Navarro et al., 2006; Chen et al., 2007; Wang et al., 2007). Some studies have documented essential roles of auxin in pathogenesis by various pathogens including *Pseudomonas syringae* DC3000 (PtoDC3000) and *P. syringae* pv. maculicola ES4326 (PmaES4326) strains (Navarro et al., 2006; Chen et al., 2007; Wang et al., 2007; Mutka et al., 2013). Other studies showed that pathogen effectors can disturb auxin physiology or signaling to affect plant responses to pathogen infection (Navarro et al., 2006; Chen et al., 2007). Auxin promotes susceptibility to pathogens via an antagonist mechanism of auxin and SA in plant defense (Wang et al., 2007; McClerklin et al., 2018). However, Mutka et al. (2013) reported that auxin promotes susceptibility to *P. syringae* via a mechanism independent of suppressing SA-mediated defenses. Thereby, it is necessary to further explore how auxin participates in plant response to infection with various pathogens.

Auxin promotes pathogen susceptibility in plants possibly due to interference of host auxin signaling and physiology by pathogens. The auxin signaling pathway is short. Auxin and its receptors, transport inhibitor response 1 (TIR1)/auxin signaling F-box (AFBs), promote degradation of auxin/indole acetic acid transcriptional repressors (AUX/IAAs) by ubiquitination, which releases auxin response factors (ARFs) to activate expression of auxin-responsive genes (Mockaitis and Estelle, 2008). Previous studies reported that *P. syringae* effector protein AvrRpt2 interferes with plant auxin signaling by promoting the degradation of AUX/IAA proteins, resulting in disease development (Navarro et al., 2006; Chen et al., 2007; Cui et al., 2013). Navarro et al. (2006) reported that *P. syringae* infection stabilizes host AUX/IAA proteins and downregulates auxin signaling to increase plant basal defense. These reports suggest that inhibiting auxin signaling may increase plant defense against bacterial pathogens, but the function of auxin in plant response to fungal pathogens remains to be explored.

The TIR1 protein, a classic receptor of auxin, is an F-box protein part of the E3 ubiquitin ligase complex SCFTIR1/AFB that mediates Aux/IAA degradation (Dharmasiri et al., 2005; Maraschin Fdos et al., 2009). TIR1 thus functions directly in plant growth and development (Tan et al., 2007). For example, in Arabidopsis, TIR1 promotes lateral root development via NAC1 transduction (Xie et al., 2000). The TIR1 interaction with indole-3-acetic acid (IAA) is stimulated by nitric oxide to modulate auxin dependent gene expression (Terrile et al., 2012). TIR1 function in auxin-related gene expression is generally negatively regulated by the miR393 involved in plant development (Sunkar and Zhu, 2004; Chen et al., 2011, 2015; Ding et al., 2013; Li and Zhang, 2016) and in response to pathogen attack (Navarro et al., 2006; Llorente et al., 2008; Jin et al., 2016; Su et al., 2021). TIR1 was reported to participate in plant response to pathogens in several studies. Navarro et al. (2006) reported that TIR1 mRNA levels were reduced by 2- to 3-fold under action of miR393 in response to pathogen attack. Overexpressing TIR1 increased tomato plant susceptibility to the *P. syringae* pv. Tomato DC3000 strain (Llorente et al., 2008). *Oryza sativa* TIR1 was stimulated by rice dwarf virus to inhibit OsIAA10 degradation, affecting plant growth and defense (Jin et al., 2016). Su et al. (2021) reported that disrupting *TaTIR1* expression increased wheat resistance to *Fusarium graminearum* infection. The reported TIR1 function in plant defense associated with miR393 reveals that auxin participates in plant immunity to pathogens partially due to TIR1 perception, but it is necessary to further explore miR393-TIR1 module function in plant defense.

Cotton is an important cash crop worldwide, and provides natural fiber as material for the textile industry, oil as a food additive, and protein as feedstuff (Ruan, 2013; Fang et al., 2017; Zhang, 2019). However, cotton plants experience various biotic stresses including pests and pathogens, which lead to great loss of yield and reduced fiber quality (Sattar et al., 2013; Phillips et al., 2017). Among these biotic stresses, Verticillium wilt mainly caused by *Verticillium dahliae* and *V. albo-atrum* seriously damages cotton production in many areas (Shaban et al., 2018; Zhang et al., 2021). *V. dahliae* is a soil-born fungus that infects plant vascular tissue, and has spores that can survive in soil for dozens of years (Kombrink et al., 2017; Qin et al., 2020; Wang et al., 2021). Verticillium wilt is difficult to control in cotton because of pathogen characteristics and a shortage of disease-resistant cotton varieties (Li et al., 2019; Billah et al., 2021). Thereby, it is vital to search for resistant genes that can be used to breed disease-resistant cultivars to control Verticillium wilt.

In this study, we addressed the function of a ghr-miR393-GhTIR1 module in the response of cotton (*Gossypium hirsutum*) to *V. dahliae* infection. GhTIR1 mRNA targeted by ghr-miR393 was cleaved through a post-transcription process based on analysis of mRNA degradome, GUS fusion reporter, and 5′-RACE. Knockdown of ghr-miR393 increased plant susceptibility to *V. dahliae* infection, while overexpression of ghr-miR393 enhanced plant resistance. Knockdown of GhTIR1 increased plant resistance against this fungal pathogen as did overexpression of ghr-miR393. Application of external IAA and PEO-IAA [2-(1H-Indol-3-yl)-4-oxo-4-phenyl-butyric acid, an auxin antagonist] showed that IAA increased plant susceptibility to *V. dahliae* infection via GhTIR1 perception. Further research revealed that GhTIR1 specifically interacts with GhIAA14 in the IAA signal pathway to participate in plant response to *V. dahliae* infection. Synergistic knockdown of *GhTIR1* and *GhICS1* in virus-induced gene silencing (VIGS) plants resulted in increased susceptibility to pathogen infection compared to the control, suggesting that GhTIR1 knockdown increasing plant resistance to *V. dahliae* depends on the SA defense pathway. qPCR and RNA-sequencing revealed that GhTIR1 knockdown promotes expression of SA-related genes. These data demonstrated that ghr-miR393-GhTIR1 module regulates plant resistance to *V. dahliae* via auxin perception, signaling pathway and the SA defense pathway.

## Materials and Methods

### Plant Materials and Cultivation Methods

Upland cotton (*Gossypium hirsutum* JM11) was grown in pots containing vermiculite. The temperature was kept constant at 28°C. The growth cycle was 16 h of light and 8 h of darkness. Cotton plants were used for virus-induced gene silencing (VIGS) analysis and *V. dahliae* infection.

Tobacco (*Nicotiana benthamiana*) seedlings were grown in a plate of vermiculite with nutritious soil at 25°C. The light/dark cycle was 16/8 h. Tobacco plants were used for GUS staining and 5′-RACE experiments as well as subcellular localization, GUS, and bimolecular fluorescence complementation (BiFC).

### Fungi Culture and Infecting Plants

*Verticillium dahliae* strain Vd080 was provided by Professor Heqin of the Institute of Cotton Research, Chinese Academy of Agricultural Sciences. It was cultured on potato dextrose agar (PDA) plates at a constant temperature of 25°C in a dark incubator. Grown hyphae were put into Czapek’s medium for 4 days, until a fungal conidia suspension concentration of 1 × 10^6^ conidia/mL was reached. Cotton plants were grown until the two leaf stage, then each cotton plant was treated with 10 ml conidia suspension.

### RNA Extraction and Real-Time Quantitative PCR (qPCR)

Total RNA from cotton samples was extracted using a plant total RNA extraction kit (Tiangen Biotech, Bejing) according to the kit instructions. First-strand cDNA was synthesized using the PrimeScript Rt reagent Kit with gDND Eraser reagent (Takara Biotech, Beijing) to analyze the expression levels of related genes by qRT-PCR. qRT-PCR was performed using MonAmp SYBR Green qPCR Mix Kit (Monad Biotech, Wuhan). To normalize gene expression, *GhUB-7* was used as an internal control.

For miRNA quantification, total RNA reverse transcription was initiated using the miRcute Plus miRNA First-Strand cDNA Kit (Tiangen Biotech, Bejing). qRT-PCR was performed using the miRcute Plus miRNA qPCR Kit (Tiangen Biotech, Bejing). To normalize gene expression, U6 snRNA was used as an internal control. All of the primers are listed in Supplementary Table 1.

### Vector Construction

Construction of the TRV-related vectors was performed as described by Liu et al. (2004) and Sha et al. (2014). The TRV vector was cut by two restriction enzymes (*Xba*I and *Bam*HI). A small tandem target mimic (STTM) sequence containing two imperfect ghr-miR393 binding sites with the same two restriction enzymes was connected to the vector. The cotton MIR393 gene was cloned from the genome and connected with TRV vector to construct the ghr-miR393-overexpressed vector (TRV:OE393). The same method was used to construct TRV:TIR1. The *GhTIR1* gene was cloned from the cotton genome and connected with TRV vector to construct the GhTIR1-silenced vector (TRV:TIR1). The same vector construction method was used to construct *GhIAA14*.

The PBI121 vector was cut by two restriction enzymes (*Xba*I and *Xma*I). *GhTIR1* was fused with the GUS gene based on the pBI121 vector, generating the pBI121-GhTIR1-GUS vector. *GhTIR1* was obtained by PCR methods and fused into GUS as a reporter, referred to as pBI121-GhTIR1^mu^-GUS. The PBI121 vector was cut by two restriction enzymes (*Bam*HI and *Sac*I). The pre-miR393 sequence was inserted into the pBI121 vector instead of the GUS gene, to construct pBI121-pre-miR393. All the plasmids were transformed into GV3101. All of the primers are listed in Supplementary Table 1.

### *Agrobacterium*-Based Invasive Plants

All carriers of construction vectors were cultured in Luria Bertani (LB) medium with 50 μg/mL kanamycin and 50 μg/mL rifampicin at 28°C overnight. *Agrobacterium* cells were harvested and then resuspended in MMA solution (10 mM MgCl_2_, 10 mM 2-(*N*-morpholino) ethanesulfonic acid (MES), and 200 μM acetosyringone; OD600 = 1.8). *Agrobacterium* cell vectors were equally mixed with auxiliary vector pYL192 for 3 h in the dark. The mixed *Agrobacterium* cells were injected into the fully expanded cotyledons of cotton seedlings with a syringe. The experiment was performed three times. All of the primers are listed in Supplementary Table 1.

### Disease Index

The disease index (DI) was calculated according to the method reported by Wang et al. (2004) as follows: DI = [(Σ disease grades × number of infected plants)/(total checked plants × 4)] × 100. The cotton seedlings were divided into five grades (0, 1, 2, 3, and 4) based on the severity of *V. dahliae* infection.

### Phylogenetic Analysis

The TIR1s in this study were retrieved from the NCBI database and the Cotton Functional Genomics Database. Neighbor-joining phylogenetic trees were constructed in MEGA7^1^ with 1,000 bootstrap replicates.

### 5′-RNA Ligase Mediated Rapid Amplification of cDNA Ends (5′ RLM-RACE)

To determine the miR393 cleavage site in TIR1, a RLM-RACE assay was performed with the RLM-RACE kit (Takara) according to the manufacturer’s instructions. Total RNA was extracted from cotton leaves, then 2 μg RNA was ligated to the RNA Oligo adaptor and used to synthesize cDNA using M-MLV reverse transcriptase according to the kit instructions. After two rounds of nested PCR experiments, the PCR products were used for sequencing. All of the primers are listed in Supplementary Table 1.

### Histochemical Staining Assay

Tobacco leaves were held in the dark for 1 day and then illuminated for 2 days. The leaves were removed and β-Glucuronidase (GUS) staining was performed. Leaves were soaked in 95% (v/v) cold acetone at 4°C overnight, then washed twice with PBS buffer. Then, leaves were soaked with staining buffer containing X-Gluc overnight at 37°C. The stained leaves were destained in absolute ethanol and 75% (v/v) ethanol. Quantification of GUS activity was performed as described by Jefferson et al. (1987).

### Hormone Treatment

Cotton phenotypes were visualized by applying the exogenous hormones IAA and PEO-IAA. Cotton plants tested for silencing efficiency after VIGS were sprayed once before *V. dahliae* infection with 5 mg/L IAA solution and 10 μM PEO-IAA solution. Cotton plants were sprayed twice after *V. dahliae* infection.

### Subcellular Localization

To determine the subcellular localization of GhTIR1, tobacco leaves were injected with *Agrobacterium* containing GhTIR1-GFP vector with H_2_B marker for 2 days in the dark. Then, tobacco leaves were visualized using a laser scanning confocal microscope (OLYMPUS FV1200). The subcellular localization of the GhIAA14 was observed in the same way. All information about the genes was obtained from the cotton database. All of the primers are listed in Supplementary Table 1.

### Yeast Two Hybrid Assay

To test for the interaction of GhTIR1 with GhIAA14, the coding sequences (CDS) of *GhTIR1* and *GhIAA14* were cloned to pGADT7 and pGBKT7, to obtain AD-GhTIR1 and BD-IAA14 vectors, respectively. Then, AD-GhTIR1 and BD-IAA14 vectors were transformed into Y2HGold. Transformed cells were cultured on synthetically defined (SD) medium without Leu or Trp. Then, yeast cells were screened on synthetic medium lacking Leu, Trp, Hde, and His (SD/-4). All of the primers are listed in Supplementary Table 1.

### Luc Assay and Bimolecular Fluorescence Complementation Assays

The CDS of *GhTIR1* were fused to an N-terminal part of firefly luciferase (N-terminal luciferase [Nluc]) and the CDS of *GhIAA14* were fused to C-terminal luciferase residues (C-terminal luciferase [Cluc]). Then, tobacco leaves were injected with *Agrobacterium* containing Nluc-GhTIR1 and Cluc-GhIAA14 for 2 days in the dark. Tobacco leaves were covered with luciferin (100 mM) and kept in the dark for 10 min. A low-light cooled charge-coupled device (CCD) imaging apparatus Lumazone_1300B (Roper Bioscience) was used to take pictures.

The CDS of *GhIAA14* were fused to the fragment encoding the C-terminus of YFP (cYFP-GhIAA14), and the CDS of *GhTIR1* were fused to the fragment encoding the N-terminus of YFP (nYFP-GhTIR1). Tobacco leaves were injected with *Agrobacterium* containing cYFP-GhIAA14 and nYFP-GhTIR1 vector with H_2_B marker for 2 days in the dark. Then, tobacco leaves were visualized using a laser scanning confocal microscope as described above. All of the primers are listed in Supplementary Table 1.

### Data Availability

The sequence data provided in this study can be found in the Cotton Functional Genomics Database,^2^ the National Center for Biotechnology Information Search database,^3^ and miRBase.^4^

## Results

### Cotton miR393 Responds to *Verticillium dahliae* Infection

**FIGURE 1:**
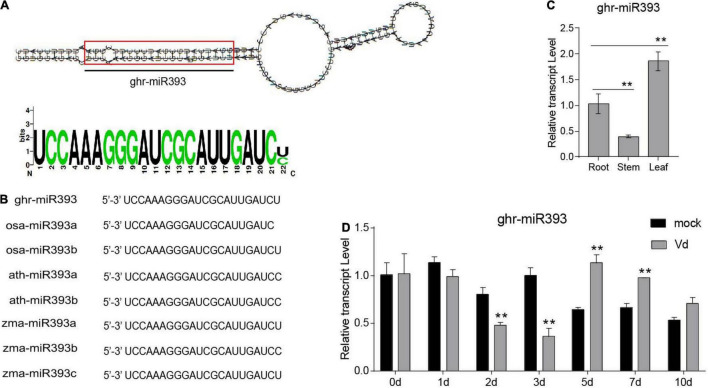
Ghr-miR393 characteristics and expression profiles. **(A)** Precursor of stem-loop structure of ghr-mir393. **(B)** The sequence alignment of ghr-mir393 and other miR393s of *Oryza sativa*, *Arabidopsis thaliana*, and *Zea mays*. **(C)** The tissue expression profile of ghr-mir393. **(D)** The expression profile of ghr-mir393 in plants inoculated with *Verticillium dahliae*. GhU6 was used as a reference gene. The error bars indicate the SD of three biological replicates. Statistical significance was determined by Student’s *t*-test, as compared with the mock treatment, **P < 0.01.

In a previous study, we identified a cotton miRNA, miR393, which later responds to *V. dahliae* infection (Hu et al., 2020a). Ghr-miR393 is a small RNA of 22 nucleotides located at the 5’-end of its precursor, in a classic stem-loop formation (Figure 1A). Ghr-miR393 contains 21 nucleotides conserved with three other species (*Oryza sativa*, *Arabidopsis thaliana*, and *Zea mays*) according to sequence alignment (Figure 1B). qPCR analysis showed that miR393 is constitutively expressed in cotton root, stem, and leaf, but preferentially in leaf (Figure 1C). To further verify whether ghr-miR393 was upregulated by *V. dahliae* infection as shown by small RNA sequencing in our previous study (Hu et al., 2020b), its expression level in plant roots infected with pathogen or mock overtime were measured by qPCR. The expression levels of ghr-miR393 increased in infected roots at 5 and 7 days post inoculation (dpi) compared to those in roots treated with mock (Figure 1D), consistent with small RNA-seq analysis (Hu et al., 2020b). Notably, ghr-miR393 expression levels in roots infected with *V. dahliae* at 2 and 3 dpi were significantly lower than those in mock roots (Figure 1D). These data demonstrated that ghr-miR393 responds to *V. dahliae* infection at later stages when the fungal pathogen is located in vascular tissue of roots.

### Identification of ghr-miR393 Target and GhTIR1 Characterization

**FIGURE 2:**
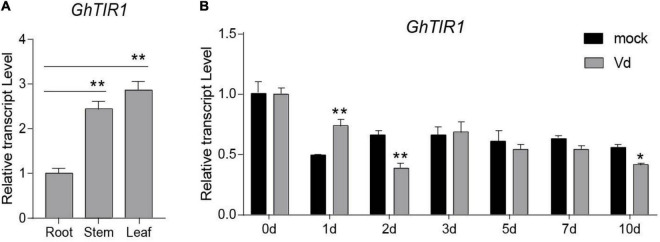
Tissue expression profile and pathogen-induced expression profile of GhTIR1. **(A)** The tissue expression profile of *GhTIR1*. **(B)** The expression profile of *GhTIR1* in plants inoculated with *Verticillium dahliae*. *GhUB-7* was used as a reference gene. The error bars indicate the SD of three biological replicates. Statistical significance was determined by Student’s *t*-test, as compared with the mock treatment, *P < 0.05, **P < 0.01.

To explore the mechanism of cotton miR393 function in plant defense, we searched for its targets according to our previous small RNA-seq (Hu et al., 2020a). In total, there were ten target genes to be predicted, which belong to TIR1, AFB2, and AFB3 (Supplementary Figure 1). According to previous mRNA degradome data, Gh_A08G1014 and Gh_D08G1288 were identified as targets of ghr-miR393, which are both noted as GhTIR1. Gh_A08G1014 and Gh_D08G1288, located in the A- and D-subgenomes, respectively, can be regarded as one gene due to high homology in nucleotides and amino acids as shown in Supplementary Figures 2, 3. GhTIR1 contains a conserved F-box domain and leucine-rich repeats (LRRs), indicating that GhTIR1 is possibly required to accept auxin molecules (Supplementary Figure 4). A phylogenetic tree showed that GhTIR1 has a highly conserved function between species (Supplementary Figure 5). GhTIR1 is constitutively expressed in root, stem, and leaf according to qPCR analysis (Figure 2A), which is not related to ghr-miR393 accumulation in various tissues.

To investigate whether GhTIR1 participates in the plant response to *V. dahliae* infection, we measured its expression levels in infected plants over 10 days by qPCR analysis. The expression levels of *GhTIR1* in plants infected with *V. dahliae* significantly increased at 1 dpi compared to those in mock treatment plants, while expression levels were significantly reduced at 2 and 10 dpi (Figure 2B), suggesting that GhTIR1 participation in plant defense is a sophisticated process. Additionally, the *GhTIR1* expression trend was contrary with ghr-miR393 accumulation (compare Figures 1D,2B), suggesting that ghr-miR393 and GhTIR1 could be a module that participates in plant response to *V. dahliae* infection.

**FIGURE 3:**
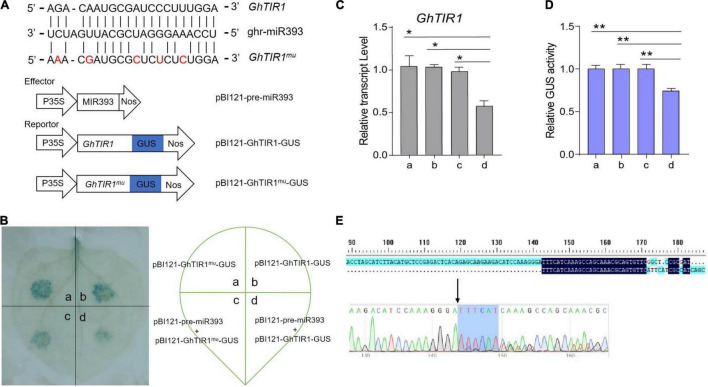
Analysis of directed *GhTIR1* degradation by ghr-miR393 via a post-transcriptional process. **(A)** Construction of the effector and reporter vectors. Red letters represent the bases mutated without changing the amino acid sequence. **(B)** GUS staining with different vectors. **(C)** Relative expression of *GhTIR1* from panel **(B)**. **(D)** Relative GUS activity from panel **(B)**. **(E)** The 5′ RLM-RACE analysis of PCR products. The error bars indicate the SD of three biological replicates. Statistical significance was determined by Student’s *t*-test: *P < 0.05, **P < 0.01.

Our previous mRNA degradome data showed that *GhTIR1* mRNA is directedly cleaved at nt 1,531 by ghr-miR393 (Supplementary Figure 6). To verify this, we performed a GUS fusion reporting assay in tobacco leaf cells. Cotton pre-miR393 sequence was inserted into a plant expression vector under control of the CaMV35S promoter as an effector, creating pBI121-pre-miR393. The CDS of *GhTIR1* and miR393-resistant *GhTIR1* (GhTIR1^mu^ mutated at complementary nucleotides with miR393) were respectively fused into *GUS* at the 5’ terminal end as reporters, referred to as pBI121-GhTIR1-GUS and pBI121-GhTIR1^mu^-GUS (Figure 3A). The tobacco leaf spots injected with pBI121-GhTIR1-GUS or pBI121-GhTIR1^mu^-GUS by needle-free syringes showed normal blue with GUS staining. When leaves were co-injected with pBI121-pre-miR393 and pBI121-GhTIR1-GUS, injected spots exhibited a slight blue, suggesting that *GhTIR1* mRNA could be largely degraded under miR393. As expected, the leaf spots co-injected with pBI121-pre-miR393 and pBI121-GhTIR1^mu^-GUS still presented as normal blue, indicating that miR393-resistent *GhTIR1* mRNA cannot be directly cleaved (Figure 3B). The results demonstrated that *GhTIR1* mRNA can be post-transcriptionally processed under the guide of ghr-miR393. Consistent with this result, the expression levels of *GhTIR1* (Figure 3C) and GUS activities in various treated leaf spots showed similar results to GUS staining (Figure 3D). Supporting this result, 5’-RACE analysis showed that *GhTIR1* mRNA was directedly cleaved at nt 1,531 (Figure 3E). The data demonstrated that ghr-miR393 and *GhTIR1* form a module that may regulate plant response to *V. dahliae* infection.

### Cotton miR393-TIR1 Module Acts in Plant Defense Against *Verticillium dahliae* Infection

**FIGURE 4:**
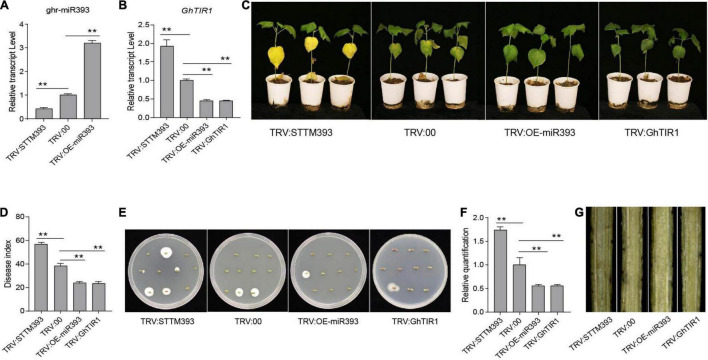
The cotton miR393-GhTIR1 module mediated plant resistance against *Verticillium dahliae*. **(A)** Relative expression of ghr-miR393 in the TRV:STTM393 and TRV:OE-miR393 plants compared to TRV:00 plants. **(B)** Relative expression of *GhTIR1* in the TRV:STTM393, TRV:OE-miR393, and TRV:GhTIR1 plants compared to TRV:00 plants. **(C)** Disease symptoms of plants at 21 dpi. **(D)** The disease index of the plants at 21 dpi. **(E)** Fungal recovery assays. Stems from cotton plants at 21 dpi were placed on PDA. Photos were taken after 5 days. **(F)** The relative *V. dahliae* fungal biomass in the infected stems was quantified by qRT-PCR. *GhUB-7* and *V. dahliae* β-tubulin were used as reference genes. **(G)** Longitudinal sections of cotton stems at 21 days. The error bars indicate the SD of three biological replicates. Statistical significance was determined by Student’s *t*-test: **P < 0.01.

To evaluate whether the ghr-miR393-*GhTIR1* module regulates plant response to fungal pathogen infection, we developed knockdown and overexpression ghr-miR393 plants and knockdown *GhTIR1* plants via various VIGS techniques (Yan et al., 2012; Sha et al., 2014). Ghr-miR393-silenced plants were developed via cooperation of the tobacco rattle virus (TRV) gene silencing system and short-tandem target mimic (STTM) technology. miR393-overexpressed plants were achieved through the TRV overexpression system and *GhTIR1*-silenced plants were generated according to the TRV silencing system. When a photobleaching phenotype marker appeared in emerging leaves of the phytoene desaturase (*PDS*)-silenced plants (Supplementary Figure 7), ghr-miR393 accumulation and *GhTIR1* expression levels were measured in corresponding VIGS plants by qPCR. As shown in Figure 4, compared to the control plants (agro-injected with empty vector, referred to as TRV:00), ghr-miR393 accumulation was significantly reduced by 57% in TRV:STTM393 plants and increased by 2.2-fold in TRV:OEmiR393 plants (Figure 4A). GhTIR1 expression level significantly decreased by 55% in TRV:GhTIR1 plants (Figure 4B), Additionally, the GhTIR1 expression levels in TRV:STTM393 and TRV:OEmiR393 plants showed significant changes compared to TRV:00, with 1.93- and 0.45-fold decreases, respectively (Figure 4B), indicating that ghr-miR393 contents can determine *GhTIR1* expression level through a post-transcriptional process.

The VIGS plants, including TRV:STTM393, TRV:OEmiR393, TRV:GhTIR1, and TRV:00 plants, were inoculated with *V. dahliae* using the root-dip method. TRV:STTM393 plants exhibited higher susceptibility to this fungal pathogen with more yellow leaves than the control at 21 dpi, while TRV:OEmiR393 and TRV:GhTIR1 plants showed higher resistance with less yellow leaves (Figure 4C). The DI of TRV:STTM393 plants was significantly higher than TRV:00 plants by 18%, whereas those of TRV:OEmiR393 and TRV:GhTIR1 plants showed lower DI values than the control, at 23.6% and 23.5 vs. 38%, respectively (Figure 4D). Supporting these results, the fungal pathogen recovery analysis showed that colony number from TRV:STTM393 stems was higher than the control, whereas those from TRV:OEmiR393 and TRV:GhTIR1 stems were lower (Figure 4E). In line with pathogen recovery results, the biomass of *V. dahliae* in infected TRV:STTM393 stems was significantly higher than the control, whereas that of TRV:OEmiR393 and TRV:GhTIR1 stems was significantly lower (Figure 4F). Longitudinal sections of stems from TRV:STTM393 plants showed darker color compared to the control, indicating that these plants were seriously damaged by this fungus, while those in TRV:OEmiR393 and TRV:GhTIR1 showed lighter color (Figure 4G). The results demonstrated that ghr-miR393 is a positive regulator of plant resistance to *V. dahliae* via a post-transcriptional process of *GhTIR* mRNA.

### *GhTIR1* Knockdown Affects IAA and Salicylic Acid Contents and Their Signal Pathways Under *Verticillium dahliae* Infection

**FIGURE 5:**
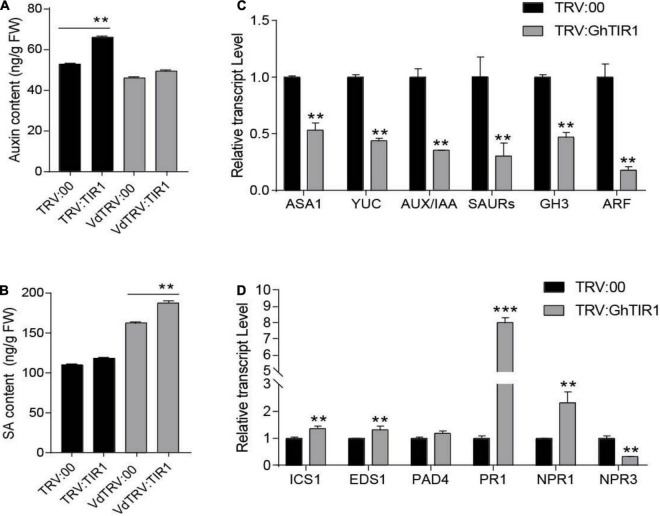
*GhTIR1* knockdown affects contents of IAA and salicylic acid (SA) and expression of their related genes. **(A)** The amount of auxin in leaves of *GhTIR1*-silenced plants compared with the control at 6 h after *Verticillium dahliae* infection. **(B)** SA content in leaves of silenced plants compared with the control at 6 h after *V. dahliae* infection. The error bars indicate the SD of three biological replicates in panels **(A,B)**. Statistical significance was determined by Student’s *t*-test: **P < 0.01. **(C)** The expressions of auxin-related genes in TRV:GhTIR1 plant roots compared with the control at 6 h after *V. dahliae* infection. **(D)** The expressions of SA-related genes in TRV:GhTIR1 plant roots compared with the control at 6 h after *V. dahliae* infection. Statistical significance as compared with TRV:00 in panels **(C,D)**, was determined by Student’s *t*-test: **P < 0.01, ***P < 0.001.

To elucidate whether GhTIR1 function in plant response to pathogen infection is involved in IAA and SA perception and signaling pathways, contents of both phytohormones and expression of their related genes were measured. HPLC-MS/MS analysis showed that IAA contents in *GhTIR1* knockdown plants were higher than the control in the resting stage (Figure 5A), consistent with research in Arabidopsis (Lakehal et al., 2019). However, under *V. dahliae* treatment, IAA contents in TRV:GhTIR1 were similar to the control. SA contents in TRV:GhTIR1 plants were similar to that in TRV:00 plants in the resting state. However, SA contents in TRV:GhTIR1 plants were higher than that in TRV:00 plants under *V. dahliae* inoculation (Figure 5B). The data demonstrated that *GhTIR1* knockdown hardly affects SA contents in the resting state, but IAA contents were higher than the control; while under *V. dahliae* infection, SA contents were significantly affected, indicating that *GhTIR1* knockdown increases SA accumulation in infected plants, but IAA contents are stable.

To investigate whether *GhTIR1* knockdown affected IAA and SA signal pathways, we measured IAA- and SA-related gene expression. We tested six IAA-related genes including two IAA biosynthesis related genes (*GhASA1* and *GhYUC*), two upregulated IAA genes (*GhSAUR* and *GhGH3*), as well as *GhIAA* and *GhARF* by qPCR analysis (Figure 5C). The results showed that expressions of six IAA-related genes were significantly reduced in TRV:GhTIR1 plants 6 h post inoculation (hpi) with *V. dahliae* compared to TRV:00 plants, indicating that *GhTIR1* knockdown decreases expressions of major IAA-related genes possibly due to feedbacks of auxin level and protein accumulation. The results of qPCR analysis showed that two SA biosynthesis-related genes, *GhICS1* and *GhEDS1*, had significantly higher expressions in TRV:GhTIR1 than TRV:00 plants at 6 hpi, indicating that *GhTIR1* knockdown promotes SA biosynthesis (Figure 5D). However, expressions of three disease resistance-related genes, *GhPR1*, *GhNPR1*, and *GhNPR3*, in TRV:GhTIR1 plants inoculated with fungal pathogen significantly changed compared to TRV:00 plants (Figure 5D). *GhPR1* and *GhNPR1* were expected to significantly increase in TRV:GhTIR1 plants compared to the control, while *GhNPR3* was significantly downregulated, suggesting that *GhTIR1* knockdown affects expression of SA response genes under *V. dahliae* infection.

### Auxin Perception Enhances Plant Susceptibility to Pathogen Infection

**FIGURE 6:**
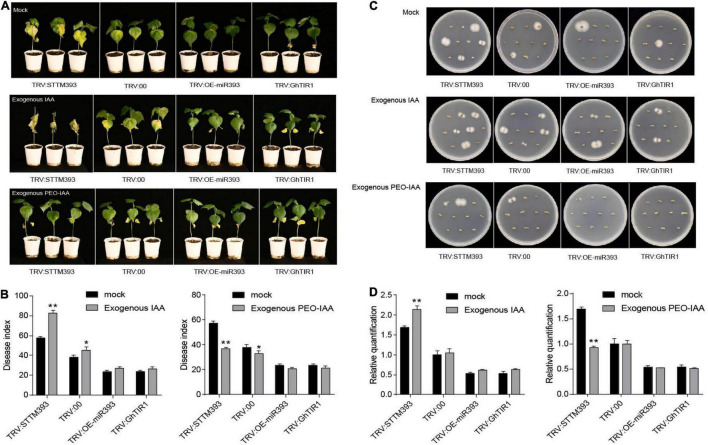
IAA promotes plant susceptibility to *Verticillium dahliae* infection. **(A)** The disease symptoms at 21 days of plants inoculated with *V. dahliae* in which exogenous IAA and PEO-IAA were applied. **(B)** The disease index of the plants at 21 dpi sprayed with exogenous IAA and PEO-IAA. Statistical significance as compared with TRV:00 was determined by Student’s *t*-test: *P < 0.05, **P < 0.01. **(C)** Fungal recovery assays. **(D)** Relative *V. dahliae* fungal biomass in the infected stems. The error bars indicate the SD of three biological replicates. Statistical significance was determined by Student’s *t*-test: **P < 0.01.

Given that *GhTIR1* knockdown increases plant resistance to *V. dahliae* infection, we further elucidated whether auxin participates in responding to the pathogen through auxin perception and signaling. Exogenous IAA and the mock were pre-applied in TRV:STTM393, TRV:OEmiR393, TRV:GhTIR1, and TRV:00 plants (the expression levels of the corresponding genes are shown in Supplementary Figure 8) before pathogen inoculation. TRV:STTM393 and TRV:00 plants showed significantly higher susceptibility to *V. dahliae* infection in exogenous IAA application with more yellow leaves at 21 dpi (Figure 6A), higher DI, and more recovering pathogen colonies and higher pathogen biomass than the mock treatment (Figures 6B–D). TRV:OEmiR393 and TRV:GhTIR1 plants with exogenous IAA application exhibited less serious symptoms than those in the mock treatment. The data suggested that IAA can promote plant susceptibility to *V. dahliae* infection depending on GhTIR1 perception of IAA.

To further evaluate GhTIR1 function in plant response to *V. dahliae* infection by perception of IAA, PEO-IAA was pre-applied in various plants including TRV:STTM393, TRV:OEmiR393, TRV:GhTIR1, and TRV:00 (the expression levels of the corresponding genes are shown in Supplementary Figure 8) before inoculation. Susceptibility of TRV:STTM393 and TRV:00 plants to *V. dahliae* infection was significantly reduced under exogenous PEO-IAA application with less yellow leaves, lower DI, and fewer recovering pathogen colonies and lower pathogen biomass than the mock treatment at 21 dpi (Figures 6A–D). TRV:OEmiR393 and TRV:GhTIR1 plants with exogenous PEO-IAA application exhibited similar symptoms compared to those in the mock treatment (Figures 6A–D). There were no significant differences in DI and pathogen biomass between PEO-IAA treatment and mock. The data from PEO-IAA treatment further suggested that IAA promotes plant susceptibility to *V. dahliae* infection at least partially due to IAA perception via GhTIR1.

### GhTIR1 Interacts With GhIAA14

**FIGURE 7:**
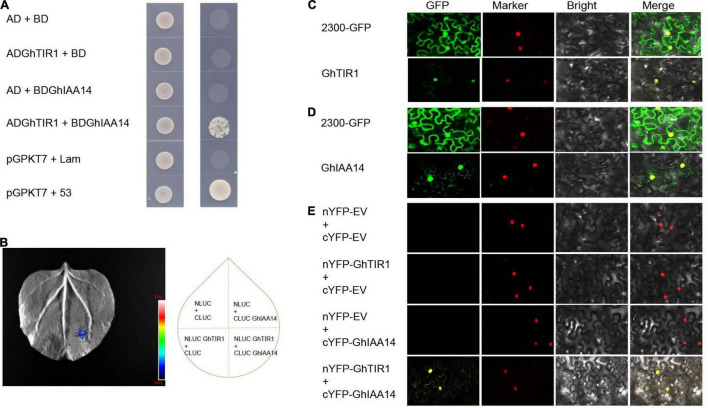
GhTIR1 interacts with GhIAA14 in the cell nucleus. **(A)** Yeast two-hybrid (Y2H) assay detection of interaction between GhTIR1 and GhIAA14. **(B)** NLUC-GhTIR1 and CLUC-GhIAA14 show LUC fluorescence. **(C,D)** Distribution of GhTIR1 and GhIAA14 in the cell. **(E)** Bimolecular fluorescence complementation (BiFC) assay showing the interaction of GhTIR1 with GhIAA14 in the cell nucleus.

It is necessary to understand the underlying mechanisms of GhTIR1 roles in plant defense through auxin perception and signal pathways. We suspected that *GhTIR1* knockdown may repress IAA degradation that does not release ARF, resulting in suppressed expression of downstream auxin-related genes. To explore the mechanism of GhTIR1 function in plant defense, we identified the GhTIR1 interacting partners. We predicted protein interactions by information analysis^5^ to identify TIR1-interacting proteins. There were ten putative interacting proteins with scores higher than 0.98 (Supplementary Figure 9). Then a yeast two hybrid assay was used to analyze which proteins interacted with GhTIR1. Only GhIAA14 interacted with GhTIR1. As shown in Figure 7A, yeasts co-transformed with BD-IAA14 vector and AD-GhTIR1 vector and positive control pGBKT7+53 can grow on SD/-Trp/-Leu/-His/-Ade medium, while yeasts co-transformed with various vector combinations including AD and BD, BD and AD-GhTIR1, BD-IAA14 and BD, and negative control pGBKT7+lam did not grow, suggesting that GhTIR1 interacts with GhIAA14 in yeast cells. To verify this interaction, we employed luciferase complementation imaging (LCI) analysis. GhTIR1 was fused into the N terminal part of LUC protein and GhIAA14 was fused into the C terminal part of LUC. As shown in Figure 7B, leaf spots agro-injected with NLUC-GhTIR1 and CLUC-GhIAA14 showed LUC fluorescence, while the spots agroinfiltrated with NLUC and CLUC, NLUC-GhTIR1 and CLUC, and NLUC and CLUC-GhIAA14 had no fluorescence (Figure 7B). These results showed that GhTIR1 can interact with GhIAA14 in plant cells.

To further explore the molecular mechanism of GhTIR1 interacting with GhIAA14, we determined the subcellular location of GhTIR1 and GhIAA14. As shown in Figure 7C, green fluorescence of GhTIR1-GFP merged with nuclear marker (H_2_B) red fluorescence, suggesting that GhTIR1 is mainly distributed in the nucleus with some in the cytoplasm, consistent with previous results in Arabidopsis (Hagen, 2015). A parallel experiment showed that GhIAA14-GFP fluorescence was distributed in the cytoplasm and the nucleus, suggesting that GhIAA14 is located in the cytoplasm and the nucleus (Figure 7D). To evaluate where GhTIR1 interacts with GhIAA14 in cells, nYFP-GhTIR1 and cYFP-GhIAA14 plant expression vectors were constructed. The tobacco mesophyll cells with co-expressed nYFP and cYFP, nYFP and cYFP-GhIAA14, and nYFP-GhTIR1 and cYFP did not present yellow fluorescence. However, yellow fluorescence appeared in mesophyll cells co-transformed with nYFP-GhTIR1 and cYFP-GhIAA14. A merged panel shows that the distribution of interaction of nYFP-GhTIR1 and cYFP-GhIAA14 was high in the nucleus and minimal in the cytoplasm (Figure 7E). The results suggested that GhTIR1 interacts with GhIAA14 mostly in the nucleus.

### Auxin Signaling Component GhIAA14 Is Involved in Plant Defense to *Verticillium dahliae* Infection

**FIGURE 8:**
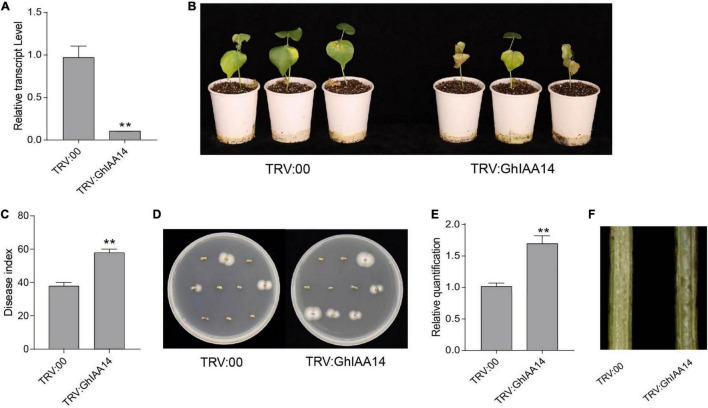
Knockdown of *GhIAA14* reduced plant resistance to *Verticillium dahliae*. **(A)** Relative expression of *GhIAA14* in the TRV:GhIAA14 plants compared to TRV:00. **(B)** Disease symptom phenotypes of the TRV:GhIAA14 plants and the control at 21 dpi. **(C)** Disease index at 21 dpi. **(D)** Fungal recovery assay. **(E)** Relative *V. dahliae* fungal biomass in the infected stems. Statistical significance was determined by Student’s *t*-test: **P < 0.01. The error bars represent the standard error of the mean of three biological replicates. **(F)** Longitudinal sections of cotton stems at 21 dpi.

Given that GhTIR1 interacts with GhIAA14, we speculated that auxin signaling component GhIAA14 could be required in the plant response to *V. dahliae* infection. We developed *GhIAA14* knockdown plants via VIGS (Figure 8A). The *GhIAA14* knockdown plants showed higher susceptibility to *V. dahliae* infection than the control with more yellow leaves (Figure 8B). In line with this result, knockdown *GhIAA14* plants showed significantly higher DI and more fungal biomass than the control (Figures 8C–F). The results demonstrated that *GhIAA14* knockdown increases plant susceptibility to *V. dahliae* partially due to auxin signaling component GhIAA14.

### *GhTIR1* Knockdown Activates the Salicylic Acid Pathway Under *Verticillium dahliae* Infection

**FIGURE 9:**
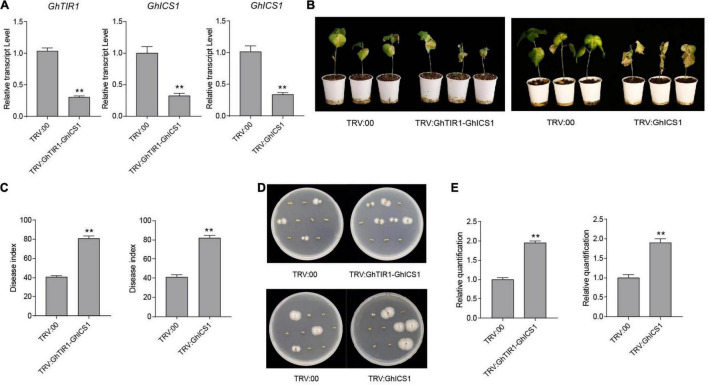
Simultaneous knockdown of *GhTIR1* and *GhICS1* reduced plant resistance to *Verticillium dahliae*. **(A)** Relative expression of *GhTIR1* and *GhICS1* in TRV:GhTIR1-GhICS1 and relative expression of *GhICS1* in the TRV:GhICS1 plants compared to TRV:00. **(B)** Disease symptom phenotypes of the TRV:GhTIR1-GhICS1 and TRV:GhICS1 plants and the control at 21 dpi. **(C)** Disease index at 21 dpi. **(D)** Fungal recovery assay. **(E)** Relative *V. dahliae* fungal biomass in the infected stems. Statistical significance was determined by Student’s *t*-test: **P < 0.01. The error bars represent the standard error of the mean of three biological replicates.

Under inoculation, SA content in *GhTIR1*-silenced plants is higher than that in the control, and expression of *GhICS1* and *GhEDS1* was significantly upregulated (Figures 5B,D). Therefore, we synergistically silenced *GhTIR1* and *GhICS1* to evaluate whether *GhTIR1* knockdown disrupted the SA pathway. The expressions of *GhTIR1* and *GhICS1* in TRV:GhTIR1-GhICS1 plants were significantly reduced by 70 and 68%, respectively, compared to the control. *GhICS1* expression level in TRV:GhICS1 was reduced by 66% (Figure 9A). Compared to the control, TRV:GhICS1 plants inoculated with *V. dahliae* showed more serious disease symptoms with more yellow leaves, higher DI, and higher fungal biomass, and synergistical knockdown of *GhTIR1* and *GhICS1* (TRV:GhTIR1-GhICS1) also showed higher susceptibility to this fungal infection (Figures 9B–E). Compared to TRV:GhTIR1 showing a disease resistance phenotype, GhICS1 is more important in plant resistance to *V. dahliae* infection, indicating that *GhTIR1* knockdown increases plant resistance partially depending on the SA defense pathway.

### Transcriptome Analysis of *GhTIR1*-Silenced Plants Compared to the Control in the Context of Fungal Pathogen Infection

To further explore the molecular mechanism of GhTIR1-silenced plant resistance, we carried out RNA-sequencing analysis between *GhTIR1* knockdown plants and the control under *V. dahliae* infection at 6 h. Fifteen seedlings were mixed as a sample to test for TRV:GhTIR1 and TRV:00 plants in triplicate. Heatmap analysis was performed using the FPKM value from three measurements.

**FIGURE 10:**
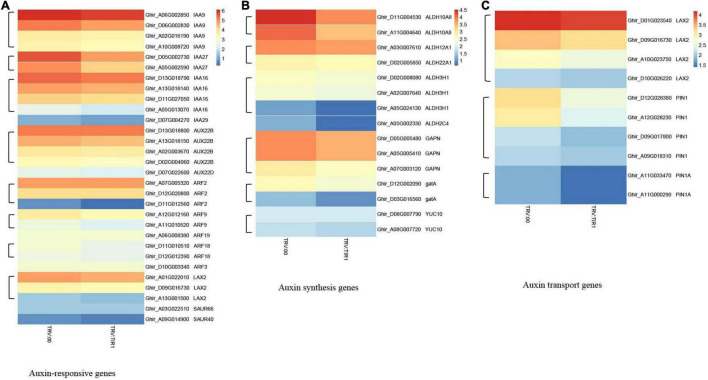
*GhTIR1* knockdown downregulated the expressions of the auxin-related genes via transcriptome analysis. Transcript contents of auxin-responsive genes **(A)**, auxin synthesis genes **(B)**, and auxin transport genes **(C)**. The FPKM value was normalized to log2 (FPKM value) for the heatmap.

**FIGURE 11:**
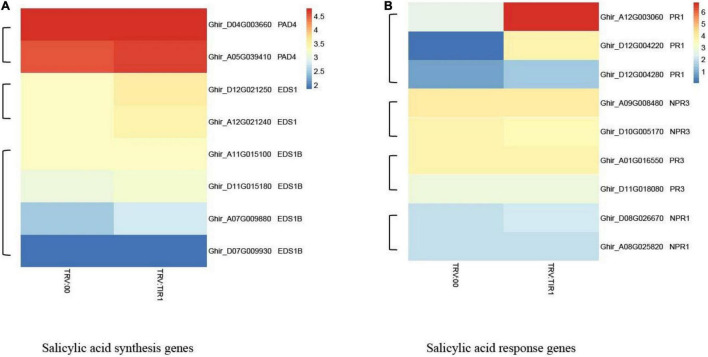
*GhTIR1* knockdown upregulated the expressions of salicylic acid pathway genes via transcriptome analysis. The expressions of salicylic acid (SA) biosynthesis genes **(A)** and SA response genes **(B)**. The FPKM value was normalized to log2 (FPKM value) for the heatmap.

The results of heatmap analysis showed that expressions of auxin-related genes, auxin synthesis genes, and auxin transport genes significantly decreased compared to the control (Figures 10A–C). However, the expressions of SA response and biosynthesis genes significantly increased (Figures 11A,B), consistent with qPCR results mentioned above. These results demonstrated that the knockdown of GhTIR1 represses IAA-related gene expression and activates SA-related gene expression, which promoted plant resistance to pathogens.

## Discussion

Auxin has been reported to participate in plant response to pathogen infection, resulting from host auxin physiology or signaling changes by pathogen challenge or pathogen auxin as well as SA resistance repression (Wang et al., 2007; Yuan et al., 2017; Tran et al., 2021). Recently, several studies have reported that auxin receptor TIR1 regulates plant immunity (Su et al., 2021), but underlying molecular mechanisms of TIR1 associated with plant response to pathogens remains mysterious. In the present study, we showed that *GhTIR1* fine-tuned by ghr-miR393 negatively regulated plant resistance against *V. dahliae* infection, resulting from auxin perception and signaling depending on the SA defense pathway.

In this study, we observed that the ghr-miR393-GhTIR1 module regulated cotton plant resistance to *V. dahliae* infection. Under *V. dahliae* treatment, ghr-miR393 accumulation was upregulated at the later stage, while GhTIR1 expression showed slight downregulation. Both *GhTIR1* knockdown and ghr-miR393 overexpression increased plant resistance to this fungal pathogen, while ghr-miR393 knockdown increased plant susceptibility. There are similar reports in tomato, rice, and wheat (Llorente et al., 2008; Jin et al., 2016; Su et al., 2021). For example, TIR1-overexpressed tomato plants showed high susceptibility to *P. syringae* infection (Llorente et al., 2008), and *TaTIR1*-silenced wheat plants exhibited high resistance to *F. graminearum* infection (Su et al., 2021). Therefore, we provide a novel example of TIR1 functioning in plant immunity, that is, the cotton miR393-TIR1 module regulates plant response to pathogen infection.

Knockdown of ghr-miR393 and the control (TRV:00) plants with pre-applied exogenous IAA have enhanced susceptibility to *V. dahliae* infection compared to the mock treatment, whereas *GhTIR1* knockdown and ghr-miR393 overexpression plants showed reduced susceptibility. However, the ghr-miR393 knockdown plants and TRV:00 plants significantly increased plant resistance with exogenous PEO-IAA compared to the mock treatment, while ghr-miR393 overexpression plants and GhTIR1 knockdown plants showed similar symptom phenotypes with PEO-IAA. These results demonstrated that IAA promotes plant susceptibility to pathogen infection via TIR1 receptor perception, which seems to be consistent with previous reports (Su et al., 2021). For instance, in *Arabidopsis thaliana* auxin is a DC3000 virulence factor that promotes pathogenicity by inhibiting host defense (McClerklin et al., 2018). These data indicate that IAA reduces plant resistance to pathogen infection partially due to IAA perception by TIR1.

Our research discovered that GhTIR1 interacts with GhIAA14 in the cell nucleus. GhIAA14 knockdown significantly decreased plant resistance to *V. dahliae* infection. These results showed that GhTIR1 acts with auxin signaling component GhIAA14 to participate in plant immunity. In Arabidopsis, the auxin response pathway was involved in plant response to *Phytophthora cinnamomi* (Eshraghi et al., 2014). In rice, auxin signaling was significantly downregulated when plants were infected by rice black streaked dwarf virus (Zhang et al., 2019). In wheat, TaTIR1 knockdown increased resistance to Fusarium head blight via disruption of the auxin signaling pathway (Su et al., 2021). Altogether, TIR1 functions in plant response to pathogen infection possibly resulting from IAA signaling disruption.

*GhTIR1* knockdown significantly increased SA content in plants inoculated with *V. dahliae* than the mock treatment. *GhTIR1*-silenced plants showed significantly higher expressions of *GhICS1*, *GhPR1*, and *GhNPR1* compared to the mock under *V. dahliae* inoculation, suggesting that *GhTIR1* knockdown increases plant resistance in part resulting from up-regulation of SA-response genes. The simultaneous knockdown of *GhTIR1* and *GhICS1* showed significant susceptibility to *V. dahliae* compared to the control. RNA-sequencing data showed that the expressions of SA related genes were upregulated in *GhTIR1* knockdown plants. These results showed that GhTIR1 participates in the plant response to pathogens depending on the SA defense pathway. Many reports supported auxin participation in plant response to pathogen infection through antagonism of SA resistance signaling. For example, in Arabidopsis, CATALASE2 activity was inhibited by SA accumulation leading to disruption of auxin biosynthesis and signaling (Yuan et al., 2017). However, some reports showed that auxin functioning in plant immunity does not depend on SA resistance signaling. For example, also in Arabidopsis, phosphite-mediated resistance may be due to stimulation of the Pi starvation responses and auxin response pathways (Eshraghi et al., 2014). In summary, GhTIR1 functioning in the plant response to *V. dahliae* partially results from repression of SA signaling by IAA perception or signaling.

## Conclusion

**FIGURE 12:**
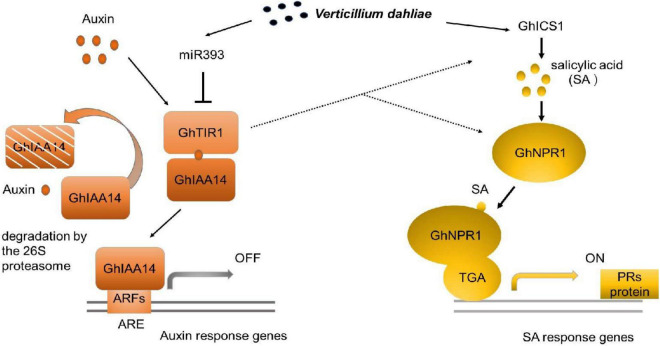
Work model of the ghr-miR393-GhTIR1 module in plant response to *Verticillium dahliae* infection. Dashed lines indicated that ghr-miR393 overexpression and *GhTIR1* knockdown activated ICS1 and NPR1 expression under *V. dahliae* infection. Auxin response element (ARE).

Our study demonstrated that *GhTIR1* expression can be fine-tuned by ghr-miR393 via a post-transcriptional process. The ghr-miR393-GhTIR1 module participates in plant resistance to *V. dahliae* infection. External IAA and PEO-IAA application reveal that IAA promotes plant susceptibility to this fungal pathogen due to GhTIR1 perception. The interaction of GhTIR1 and GhIAA14 functions in plant immunity resulting from IAA signaling disruption. Additionally, *GhTIR1* knockdown affected SA content and increased expression of SA response genes under *V. dahliae* infection. Simultaneous knockdown of *GhTIR1* and *GhICS1* increases plant susceptibility to pathogens, suggesting that GhTIR1 participates in plant resistance depending on the SA defense pathway. A simple work model of the ghr-miR393-GhTIR1 module is shown in Figure 12. Overall, we demonstrated that auxin receptor TIR1 affects auxin perception and signaling and serves as an antagonist to SA resistance signaling.

## Data Availability Statement

The datasets presented in this study can be found in online repositories. The names of the repository/repositories and accession number(s) can be found below: NCBI SRA: SRR18322379–SRR18322384. BioSample: SAMN26533846: cotton JM11 (TaxID: 34274). BioProject: PRJNA813993.

## Author Contributions

GS performed the experiments and the data analysis and wrote the manuscript. SW analyzed the data. JZ, YT, and XG conceived and designed the experiments. PW, GZ, and FL participated in the experimental design. JW conceived and designed the experiments, wrote the manuscript, and edited the manuscript. All authors read and approved the final manuscript.

## Conflict of Interest

The authors declare that the research was conducted in the absence of any commercial or financial relationships that could be construed as a potential conflict of interest.

## Publisher’s Note

All claims expressed in this article are solely those of the authors and do not necessarily represent those of their affiliated organizations, or those of the publisher, the editors and the reviewers. Any product that may be evaluated in this article, or claim that may be made by its manufacturer, is not guaranteed or endorsed by the publisher.

## Footnotes

1
https://www.megasoftware.net/

2
https://www.cottongen.org/

3
https://www.ncbi.nlm.nih.gov/

4
https://www.mirbase.org/

5
https://cn.string-db.org

## Supplementary Material

Supplementary Figure 1 Ten target genes to be predicted (phylogenetic trees were inferred with 1,000 replicates).

Supplementary Figure 2 Two nucleotide sequence alignments of Gh_A08G1014 and Gh_D08G1288.

Supplementary Figure 3 Two amino acid sequence alignments of Gh_A08G1014 and Gh_D08G1288.

Supplementary Figure 4 GhTIR contains a conserved F-box domain and leucine-Rich Repeats (LRRs).

Supplementary Figure 5 Phylogenetic trees of GhTIR1 and other plant TIR1s. Phylogenetic trees were inferred with 1,000 replicates. Sequences were deposited in GenBank (https://www.ncbi.nlm.nih.gov/genbank/) under the following accession numbers: MeTIR1 (XP_021613033), HbTIR1 (XP_021639267), JcTIR1 (XP_012082666), AtTIR1 (AT3G62980), GhTIR1 (Gh_A08G1014), AtAFB2 (AT3G26810), OsTIR1 (XP_015635915), ZmTIR1 (XP_008669494).

Supplementary Figure 6 Ghr-miR393 directed GhTIR1 cleavage at nt 1,531.

Supplementary Figure 7 Photobleaching phenotypes of GhPDS-silenced plant leaves.

Supplementary Figure 8 Relative expression levels. **(A–C)** Relative expression levels of ghr-miR393 in the TRV:STTM393 and TRV:OE-miR393 plants and GhTIR1 in the TRV:GhTIR1 plants compared to TRV:00. The error bars indicate the SD of three biological replicates. Statistical significance was determined by Student’s *t*-test: **P* < 0.05, ^**^*P* < 0.01.

Supplementary Figure 9 Protein interaction prediction. This is the result of the prediction in Arabidopsis. Comparing IAA7 protein sequence of Arabidopsis to the protein sequence of cotton, we gained IAA14.
