# Supplementary figures and images for: Cotton miR393-TIR1 Module Regulates Plant Defense Against Verticillium dahliae via Auxin Perception and Signaling
Source: Front Plant Sci. 2022 May 3;13:888703. doi: 10.3389/fpls.2022.888703 (PMC9111529; doi:10.3389/fpls.2022.888703)

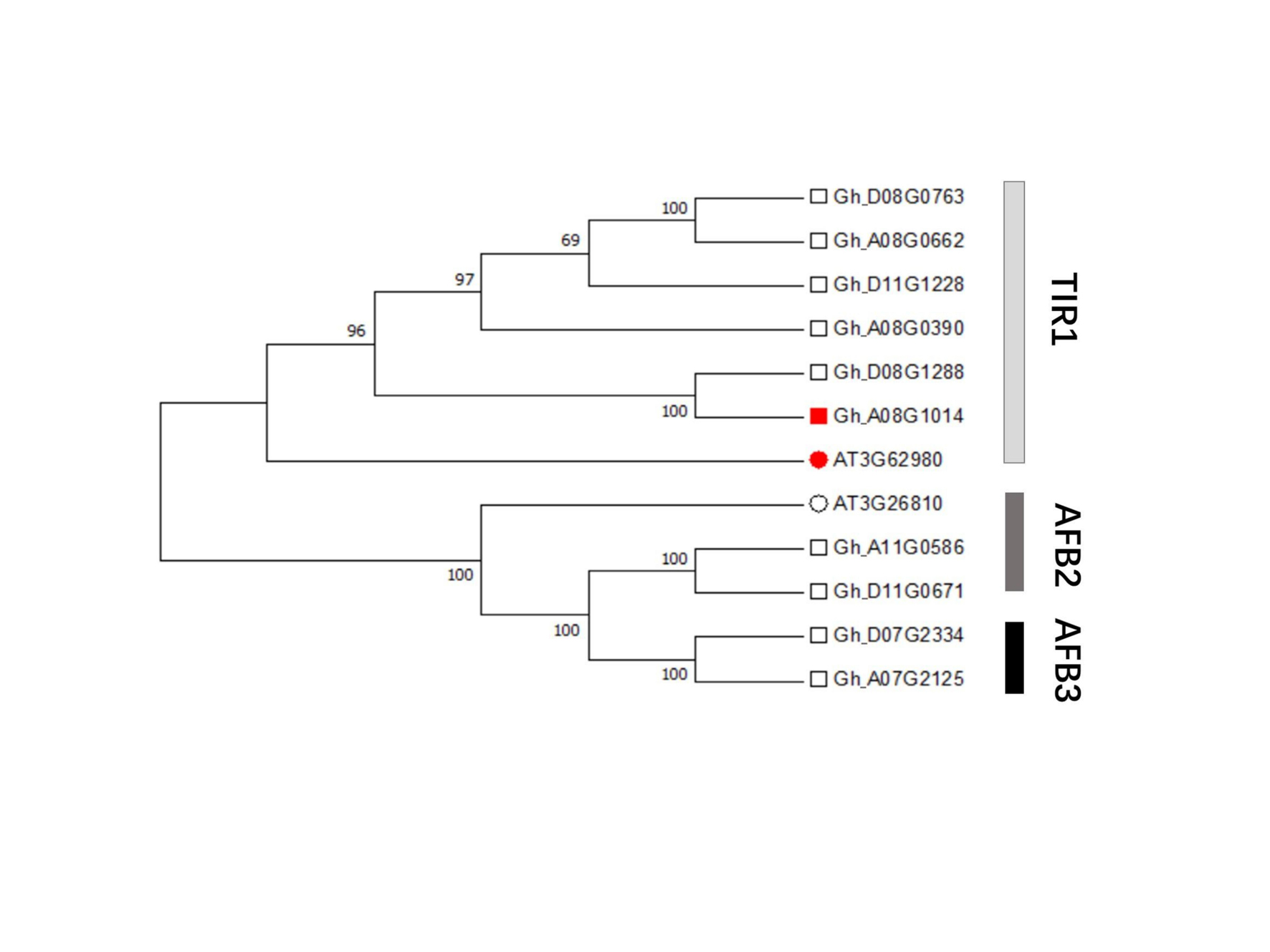

Supplement: Supplementary Figure 1 — Ten target genes to be predicted (phylogenetic trees were inferred with 1,000 replicates). [file Image_1.JPEG]

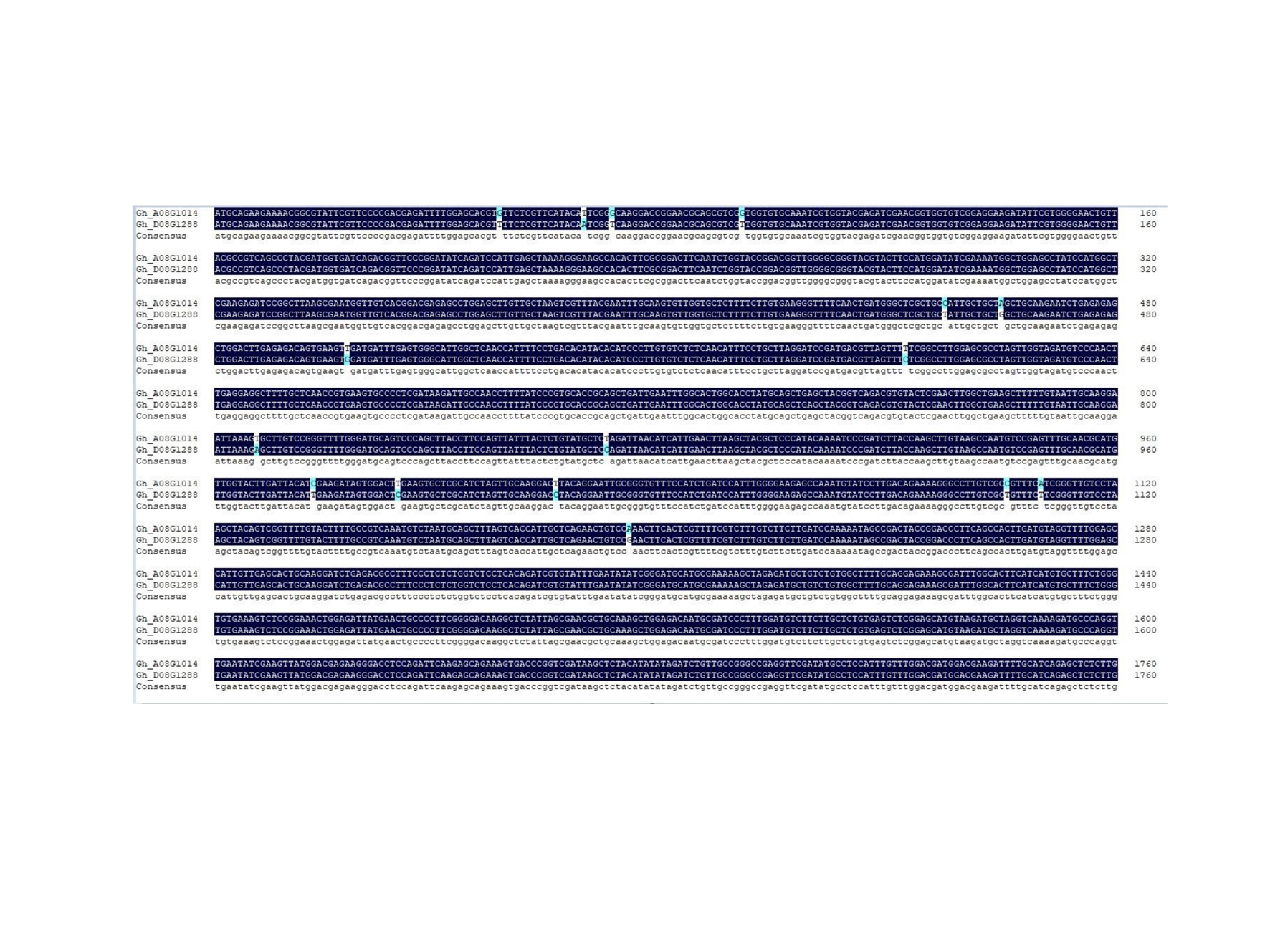

Supplement: Supplementary Figure 2 — Two nucleotide sequence alignments of Gh_A08G1014 and Gh_D08G1288. [file Image_2.JPEG]

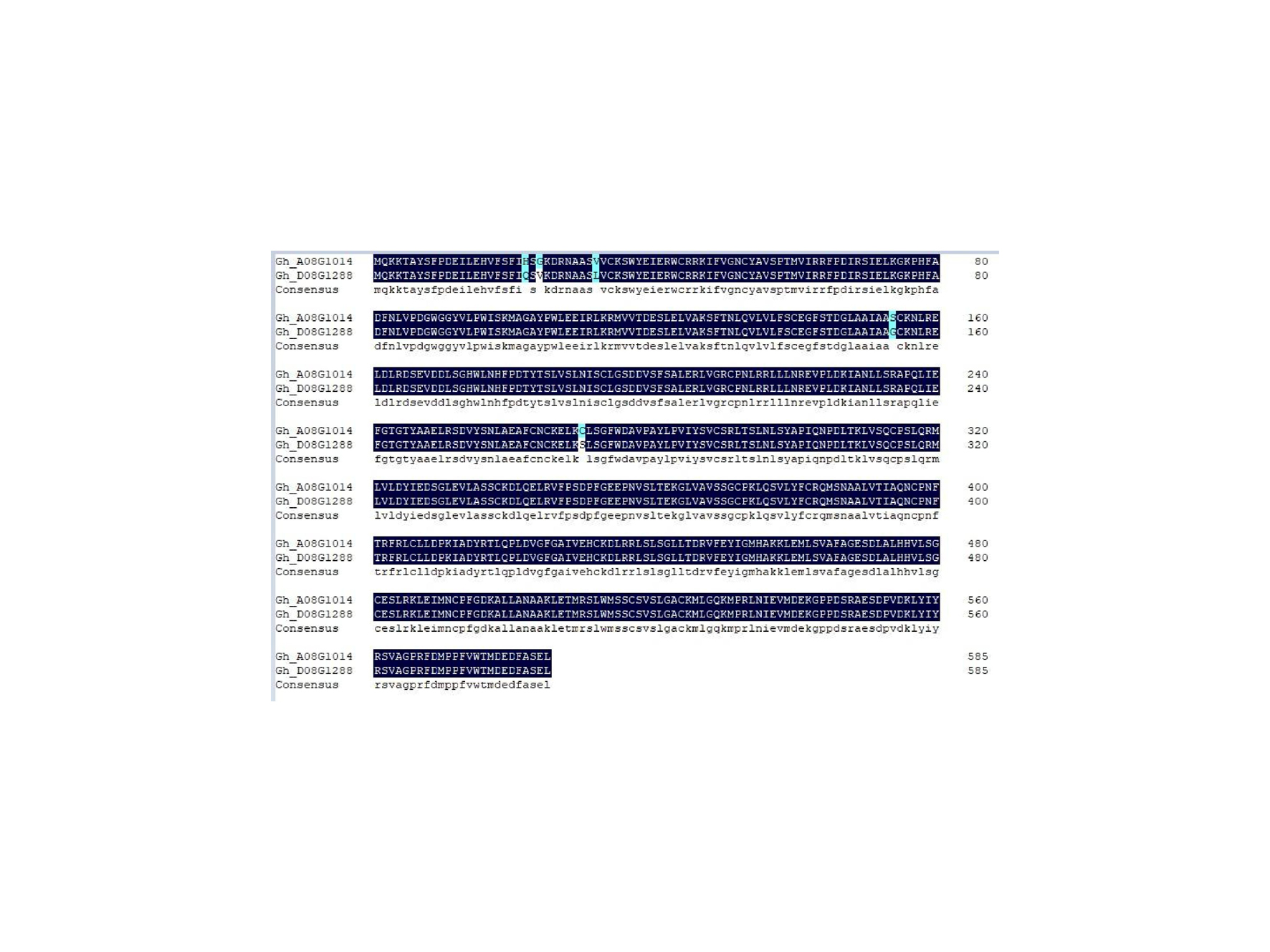

Supplement: Supplementary Figure 3 — Two amino acid sequence alignments of Gh_A08G1014 and Gh_D08G1288. [file Image_3.JPEG]

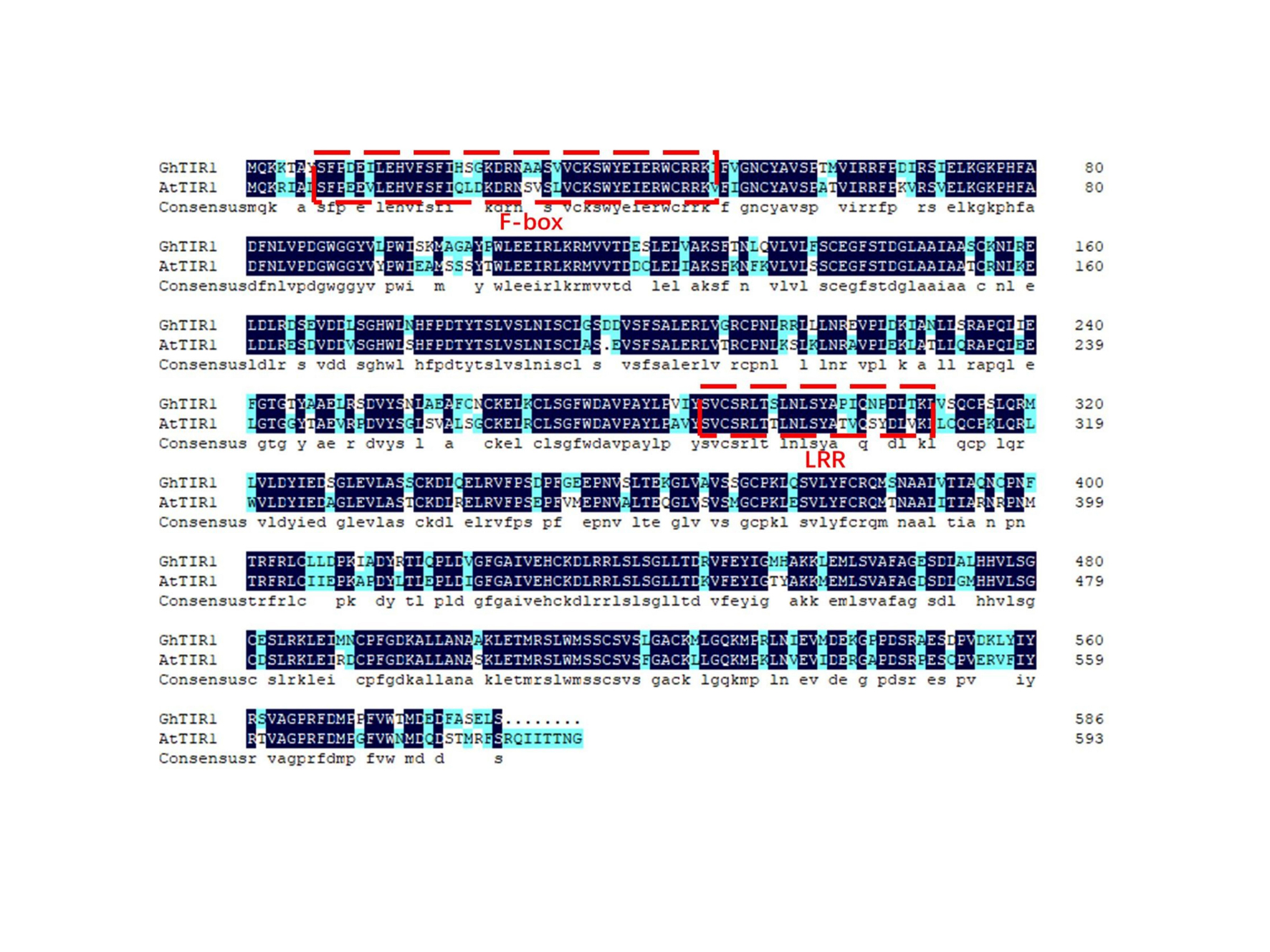

Supplement: Supplementary Figure 4 — GhTIR contains a conserved F-box domain and leucine-Rich Repeats (LRRs). [file Image_4.JPEG]

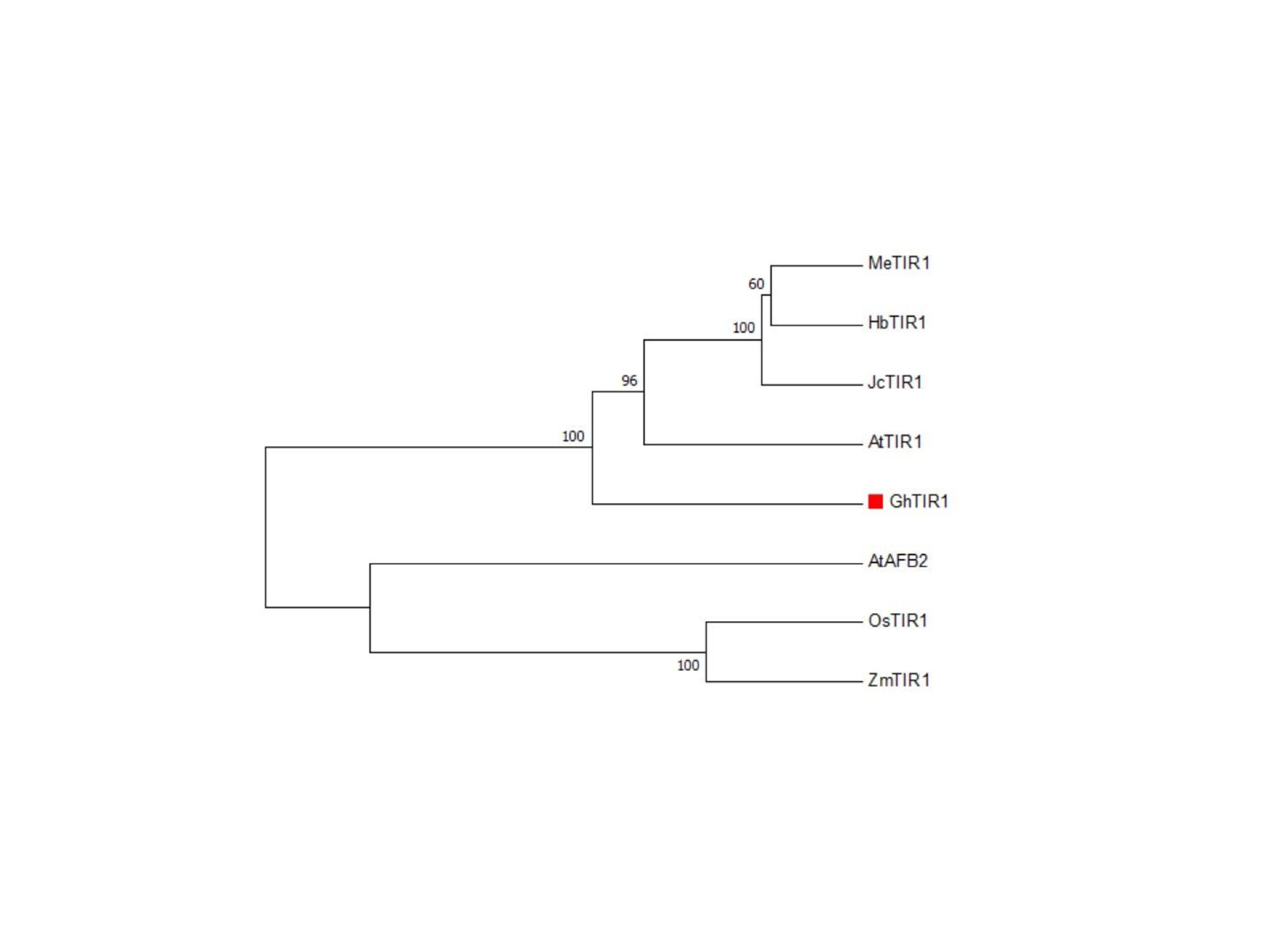

Supplement: Supplementary Figure 5 — Phylogenetic trees of GhTIR1 and other plant TIR1s. Phylogenetic trees were inferred with 1,000 replicates. Sequences were deposited in GenBank (https://www.ncbi.nlm.nih.gov/genbank/) under the following accession numbers: MeTIR1 (XP_021613033), HbTIR1 (XP_021639267), JcTIR1 (XP_012082666), AtTIR1 (AT3G62980), GhTIR1 (Gh_A08G1014), AtAFB2 (AT3G26810), OsTIR1 (XP_015635915), ZmTIR1 (XP_008669494). [file Image_5.JPEG]

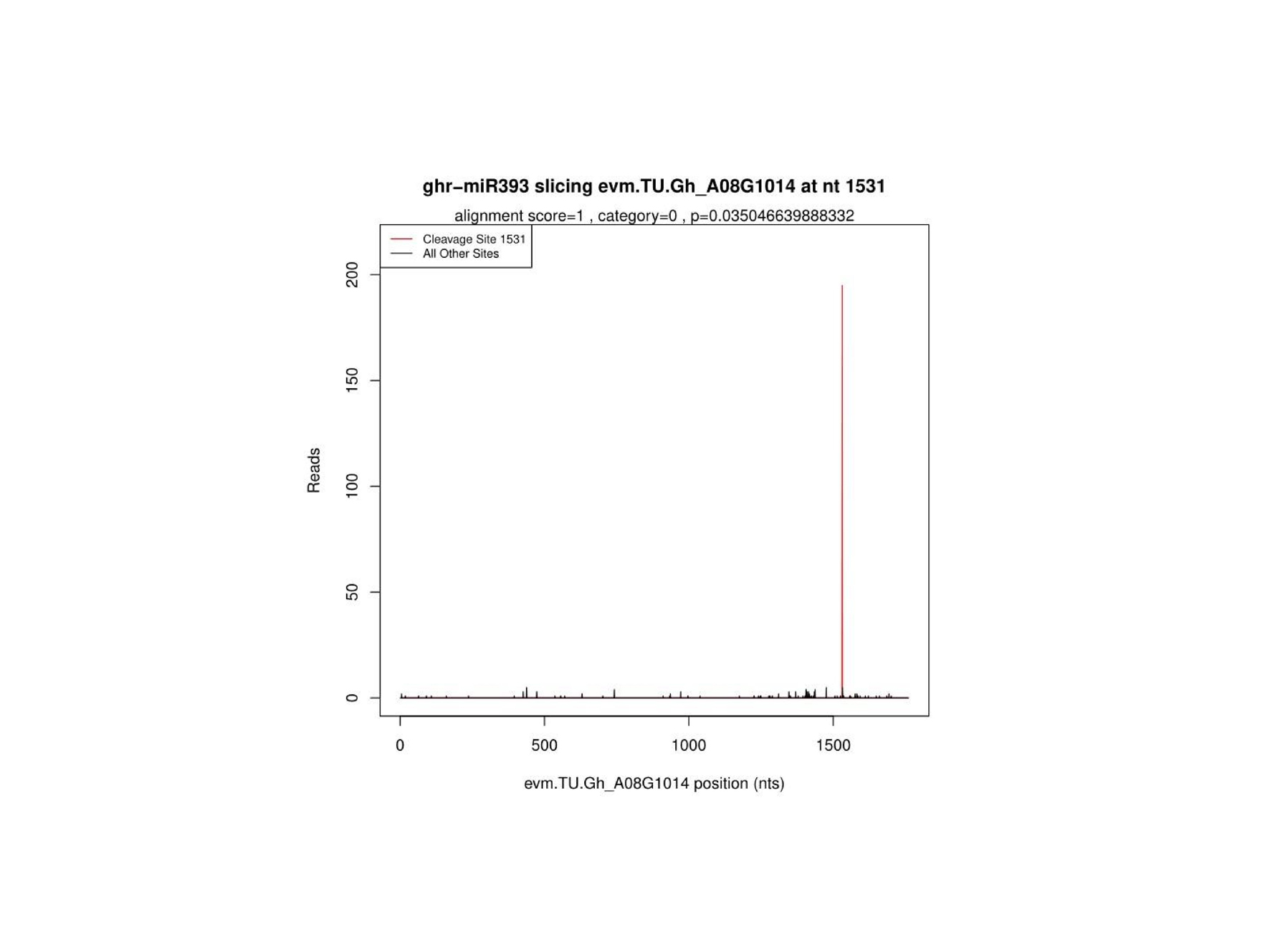

Supplement: Supplementary Figure 6 — Ghr-miR393 directed GhTIR1 cleavage at nt 1,531. [file Image_6.JPEG]

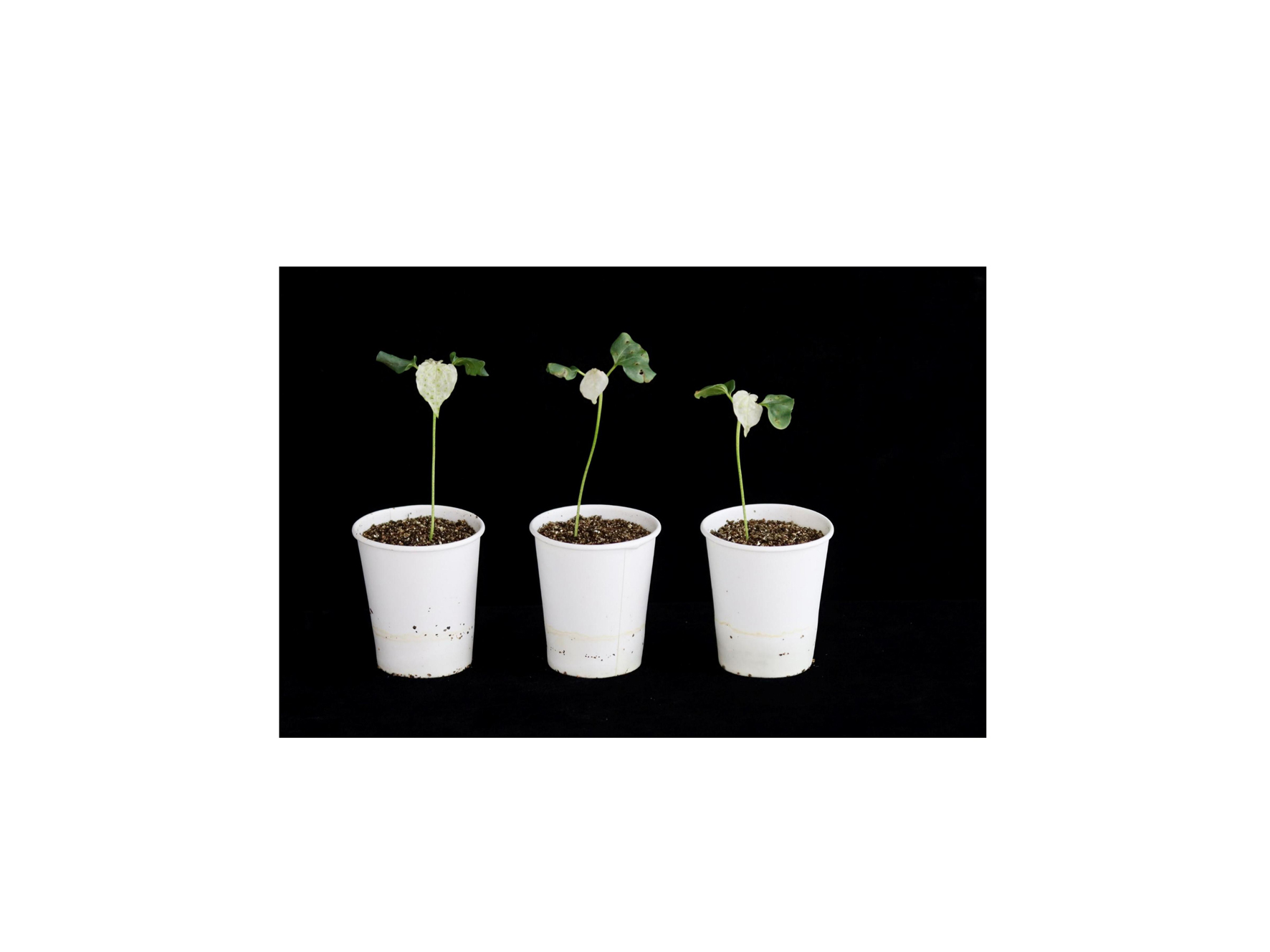

Supplement: Supplementary Figure 7 — Photobleaching phenotypes of GhPDS-silenced plant leaves. [file Image_7.JPEG]

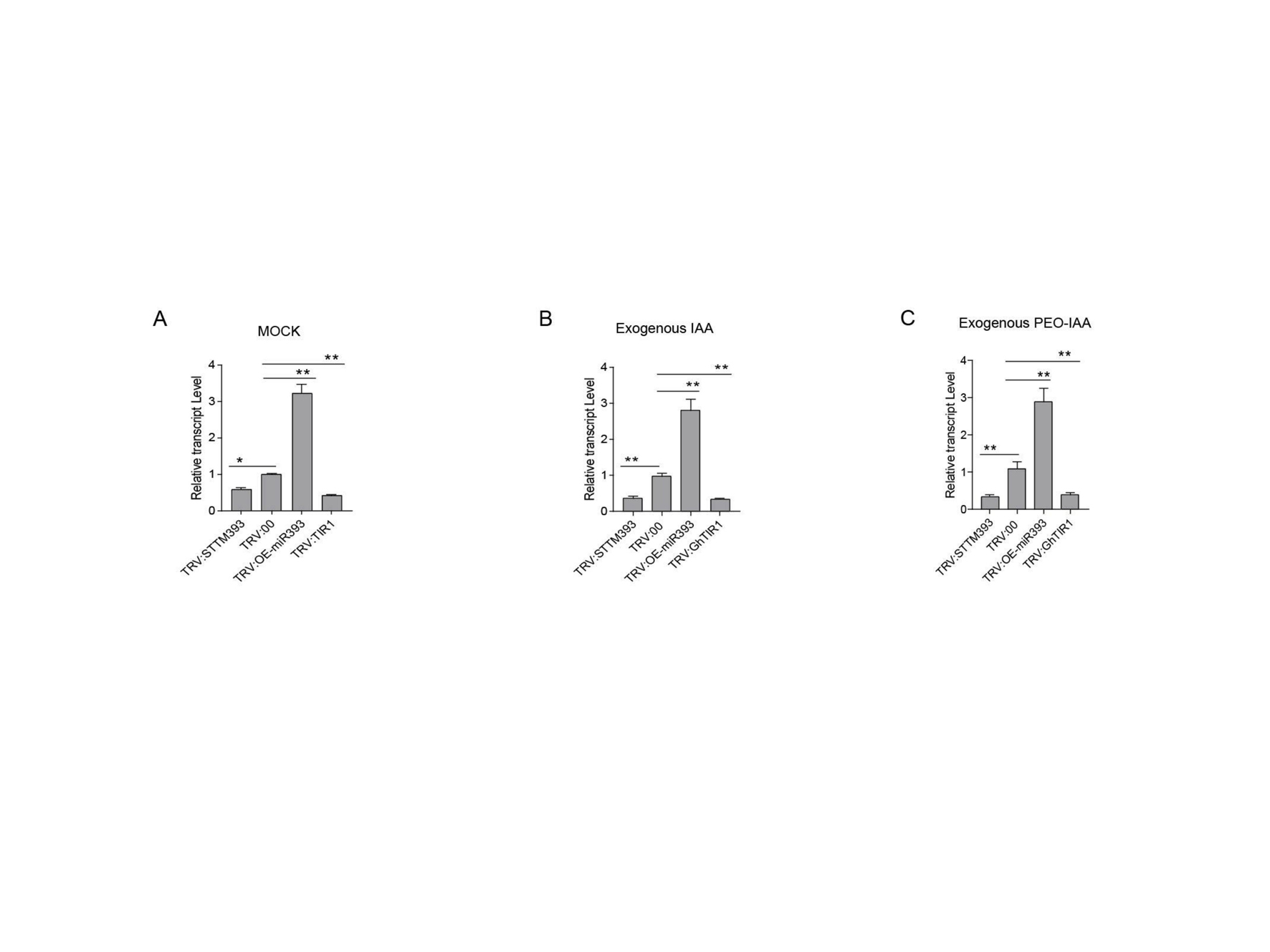

Supplement: Supplementary Figure 8 — Relative expression levels. (A–C) Relative expression levels of ghr-miR393 in the TRV:STTM393 and TRV:OE-miR393 plants and GhTIR1 in the TRV:GhTIR1 plants compared to TRV:00. The error bars indicate the SD of three biological replicates. Statistical significance was determined by Student’s t-test: *P < 0.05, **P < 0.01. [file Image_8.JPEG]

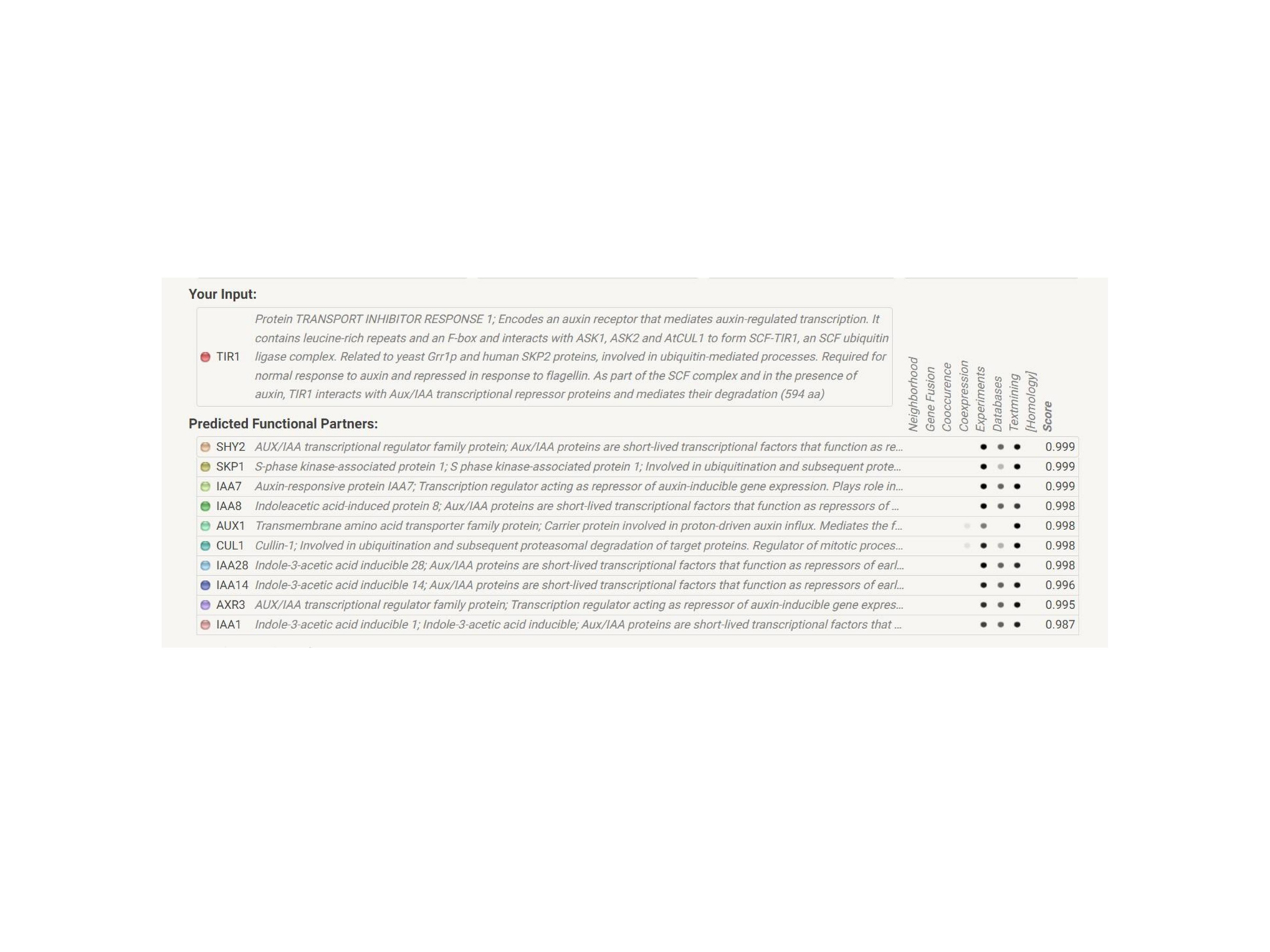

Supplement: Supplementary Figure 9 — Protein interaction prediction. This is the result of the prediction in Arabidopsis. Comparing IAA7 protein sequence of Arabidopsis to the protein sequence of cotton, we gained IAA14. [file Image_9.JPEG]
