# Supplementary material for: Cotton miR393-TIR1 Module Regulates Plant Defense Against Verticillium dahliae via Auxin Perception and Signaling
Source: Front Plant Sci. 2022 May 3;13:888703. doi: 10.3389/fpls.2022.888703 (PMC9111529; doi:10.3389/fpls.2022.888703)
Supplement: Supplementary file 10 [file Table_1.docx]

**Supplementary Table S1** The primer sequences used in this study.

| **Primer** | **Sequence（5’-3’ ）** | **destination** |
| --- | --- | --- |
| OEmiR393-F | AAGGTTACCGAATTCTCTAGAGGTGGAGAGCTCCAAAGGGATC | overexpression |
| OEmiR393-R | GAGCTCGGTACCGGATCCGATGGAAAATTCCGAAGGGATC | overexpression |
| STTM393-F | AAGGTTACCGAATTCTCTAGAAGATCAATGCGACAGTCCCTTTGGAGTTGTTGTTGTTATGGTC | STTM |
| STTM393-R | GAGCTCGGTACCGGATCCTCCAAAGGGACTGTCGCATTGATCTATTCTTCTTCTTTAGACC | STTM |
| vGhTIR1-F | AGTAAGGTTACCGAATTCACTTCCATGGATATCGAA | VIGS |
| vGhTIR1-R | GAGCTCGGTACCGGATCCGTTGAGCCAATGCCCACT | VIGS |
| pBI121-pre-miR393-F | GAACACGGGGGACTCTAGAGGATCCGGTGGAGAGCTCCAAAGGGATC | transient expression |
| pBI121-pre-miR393-R | TGAACGATCGGGGAAATTCGAGCTCGATGGAAAATTCCGAAGGGATC | transient expression |
| pBI121-GhTIR1-GUS-F | GAGAGAACACGGGGGACTCTAGAATGCAGAAGAAAACGGCGTATTC | transient expression |
| pBI121-GhTIR1-GUS-R | AACATAAGGGACTGACCACCCGGGAGAGAGCTCTGATGCAAAATC | transient expression |
| pBI121-GhTIR1^mu^-GUS-F | GAAACGATGCGCTCTCTCTGGATGTCTTCTTGCTCTGTGAGT | transient expression |
| pBI121-GhTIR1^mu^-GUS-R | CATCCAGAGAGAGCGCATCGTTTCCAGCTTTGCAGCGTTCG | transient expression |
| qU6-F | CGTCGAGCTATTTGCCGCAGGACCTC | qRT-PCR |
| qmiR393-F | CCGCGTCCAAAGGGATCGCATTGATCT | qRT-PCR |
| Gh_A08G1014-F | ACAGGAATTGCGGGTGTTTCCA | qRT-PCR |
| Gh_A08G1014-R | TAGGACAACCCGATGAAACGGC | qRT-PCR |
| GhUBQ7-F | GAAGGCATTCCACCTGACCAAC | qRT-PCR |
| GhUBQ7-R | CTTGACCTTCTTCTTCTTGTGCTTG | qRT-PCR |
| ACTF | TCCTGATGGGCAAGTGATTAC | qRT-PCR |
| ACTR | TTGTATGTGGTCTCGTGGATTC | qRT-PCR |
| V. d tubulin-F | AACAACAGTCCGATGGATAATTC | qRT-PCR |
| V. d tubulin-R | GTACCGGGCTCGAGATCG | qRT-PCR |
| qASA1-F | GTCCGTACGCTCAGATGCACTT | qRT-PCR |
| qASA1-R | TCCAACACTGGAAGCCAGCAAA | qRT-PCR |
| qYUC-F | ATCGAGCTCAAGAACGTCACGG | qRT-PCR |
| qYUC-R | TCCTTTGAGCCAAGTAGGCACG | qRT-PCR |
| qAUX/IAA-F | CTGCTCCTCCTCCTCCAAAAGC | qRT-PCR |
| qAUX/IAA-R | TAAATCCCACCACCCTCCGACT | qRT-PCR |
| qSAUR-F | GGGCATTGGATTGGTTCATGCG | qRT-PCR |
| qSAUR-R | TGTCTTCTCCGACGTAAACCGC | qRT-PCR |
| qGH3-F | AGGTCATTGTTGAGTCGCTTGGT | qRT-PCR |
| qGH3-R | CCCTTTGCTTGTGATCTCCCAGA | qRT-PCR |
| qARF-F | ACTTTCAAGCTCCTGCGCTACC | qRT-PCR |
| qARF-R | GGCCATGTCATAGGTGCCTGAG | qRT-PCR |
| qICS1-F | ATCGAGTGGCTCCATGCTCAAC | qRT-PCR |
| qICS1-R | CGGCGGCACCAACAAGATTATG | qRT-PCR |
| qEDS1-F | GCTTCCACTCTCGTCGAGTGAC | qRT-PCR |
| qEDS1-R | CCCTAGTGCTCAAGCCAAGGTC | qRT-PCR |
| q-PAD4-F | GGATGGAAGAATGGAAAGAAATGAA | qRT-PCR |
| q-PAD4-R | GAACTAGGAAAGCAGACTAAGGAACCA | qRT-PCR |
| qGhPR1-F | GGGGCAGTGCTGACCTATCG | qRT-PCR |
| qGhPR1-R | TTAGCACAACCAAGATGGACAGAGT | qRT-PCR |
| qNPR1-F | GCGAATCGGCTTTCTTCTTCA | qRT-PCR |
| qNPR1-R | CACGTGGTGCTGTTGTTGTTACTG | qRT-PCR |
| qNPR3-F | AGCTTAGAACACCTCGTTTCCGA | qRT-PCR |
| qNPR3-R | ACACCTACCGAAACACCTTCAACC | qRT-PCR |
| AD-GhTIR1-F  (Gh_A08G1014) | GCCATGGAGGCCAGTGAATTCATGCAGAAGAAAACGGCG | Yeast two hybrid |
| AD-GhTIR1-R  (Gh_A08G1014) | TGCAGCTCGAGCTCGATGGATCCCAGAGAGCTCTGATGCAAAATC | Yeast two hybrid |
| NLUC-GhTIR1-F | CACGGGGGACGAGCTCGGTACCATGCAGAAGAAAACGGCG | Luc assay |
| NLUC-GhTIR1-R | ACGCGTACGAGATCTGGTCGACAGAGAGCTCTGATGCAAAATC | Luc assay |
| nYFP-GhTIR1-F | CATCGAGGACGCCGGCGGATCCATGCAGAAGAAAACGGCGTATT | BiFC assay |
| nYFP-GhTIR1-R | GAAAGCTCTGCAGGTCGACTCTAGAAGAGAGCTCTGATGCAAAAT | BiFC assay |
| GhTIR1-GFP-F | AGAACACGGGGGACTCTAGAATGCAGAAGAAAACGGCG | subcellular location |
| GhTIR1-GFP-R | TCTCCTTTACCCATGTTAATTAAAGAGAGCTCTGATGCAAAATC | subcellular location |
| BD-GhIAA14-F  (Gohir.D09G224500) | CATATGGCCATGGAGGCCGAATTCATGGAAGTTGGTCGAAAAATG | Yeast two hybrid |
| BD-GhIAA14-R  (Gohir.D09G224500) | TGCGGCCGCTGCAGGTCGACGGGCTCTGCTCTTGCATTTC | Yeast two hybrid |
| CLUC-GhIAA14-F | TACGCGTCCCGGGGCGGTACCATGGAAGTTGGTCGAAAAATG | Luc assay |
| CLUC-GhIAA14-R | ACGAAAGCTCTGCAGGTCGACGGCTCTGCTCTTGCATTTC | Luc assay |
| cYFP-GhIAA14-F | CAATTACAGGTACCCGGGGATCCATGGAAGTTGGTCGAAAAATG | BiFC assay |
| cYFP-GhIAA14-R | CTGCCACCGCCGTCGACTCTAGAGGCTCTGCTCTTGCATTTC | BiFC assay |
| GhIAA14-GFP-F | AGAACACGGGGGACTCTAGAATGGAAGTTGGTCGAAAAATG | subcellular location |
| GhIAA14-GFP-R | TCTCCTTTACCCATGTTAATTAAGGCTCTGCTCTTGCATTTC | subcellular location |
| vGhIAA14-F | AAGGTTACCGAATTCTCTAGAAACATGCTTGGAACTGACG | VIGS |
| vGhIAA14-R | GAGCTCGGTACCGGATCCGGATCTTTGGCAGAACCTG | VIGS |
| Gohir.D09G224500-F | GGTGGAATCGCTGTTGTTGCTG | qRT-PCR |
| Gohir.D09G224500-R | TTGTGCCTTGGCAGGAGGTTTT | qRT-PCR |
| VGhICS1-F | AAGGTTACCGAATTCTCTAGAGATGAATGGGTGCGAAGG | VIGS |
| VGhICS1-R | GAGCTCGGTACCGGATCCCACTCGATTGCTTCGATTTG | VIGS |
| VGhTIR1-GhICS1-F | AAGGTTACCGAATTCTCTAGAGATGAATGGGTGCGAAGG | VIGS |
| VGhTIR1-GhICS1-R | GAGCTCGGTACCGGATCCCAAATCATCAACTTCACTG | VIGS |
| GhTIR1-GhICS1-F | CAAATCGAAGCAATCGAGTGAATGGCTGGAGCCTATCC | Nested PCR |
| GhTIR1-GhICS1-R | GGATAGGCTCCAGCCATTCACTCGATTGCTTCGATTTG | Nested PCR |
